# Synthesis of carbonyl-functionalized mercaptosilsesquioxane

**DOI:** 10.1038/s41598-025-14360-x

**Published:** 2025-08-11

**Authors:** Kamil Hanek, Patrycja Żak

**Affiliations:** Department of Organometallic Chemistry, Faculty of Chemistry, Adam Mickiewicz University in Poznań, Uniwersytetu Poznańskiego 8, 61-614 Poznań, Poland

**Keywords:** Chemistry, Catalysis, Green chemistry, Materials chemistry, Chemical synthesis

## Abstract

Commercially available and inexpensive potassium carbonate (K_2_CO_3_) has been applied to the hydrothiolation of mercaptopropylisobutyl POSS (**SQ-SH**) with α,β-unsaturated carbonyl compounds of different types. This innovative approach has been proved to be effective for a wide range of substrates, leading to novel classes of functionalized SQ-based materials in yields exceeding 90% under ambient temperature, air and transition metal-free conditions. Additionally, the proposed synthetic strategy has been used for the modification of chalcones, the compounds with significant medicinal and synthetic potential. As a result, twelve new mercapto-modified nanomaterials containing carbonyl groups were obtained and comprehensively characterized by spectroscopic methods and mass analysis. The selected product of great practical application was studied in terms of its thermal properties.

**Supplementary Information:**

The online version contains supplementary material available at 10.1038/s41598-025-14360-x.

## Introduction

One of the most important chemical structures with great application potential are α,β-unsaturated carbonyl compounds. They have been the subjects of continuous research interest as they show certain specific properties of importance for development of medicinal chemistry. For instance, they can be used in the design of new drugs because of their antibacterial, antiviral, antiallergic, antimalarial, antimicrobial, anticancer, anti-fungal and anti-inflammatory activities^1–14^. In order to improve their desirable properties and enrich their use in contemporary science some research groups set their focus on organosulfur carbonyl compounds. The presence of a sulfur heteroatom is highly desirable since the moieties containing such heteroatoms are characterized with biodegradability^15^, that is extremely important taking into account the growing market for environmentally friendly chemical products. Furthermore, the organosulfur compounds can serve as building blocks in biological systems^16^, they exhibit potential catalytic activity in cascade reactions^17^, display antiproliferative properties^18^ and they are able to act as SARS-CoV-2M^PRO^ inhibitors^19^.

Literature provides only a few methods for obtaining β-sulfenylated carbonyl compounds and most of them are based on sulfa-Michael addition (SMA) in the presence of an organocatalyst. Unfortunately, many of the methods proposed so far struggle with significant number of drawbacks, namely, they need toxic solvents, inert atmosphere, non-stoichiometric ratios of reagents, high concentrations of catalysts and long reaction time^20–23,23–27‚28^. Recently, in our research group we have discovered a simple, yet effective base-catalyzed protocol for obtaining β-sulfenylated carbonyl compounds^29^. Our protocol allowed full elimination of transition metals, solvents and any other additives, offered short reaction time, full chemoselectivity, facile isolation of the final product, thereby permitting elimination of additional operations and reduction of production cost and time. Additionally, in comparison with the hitherto available synthetic procedures, the method we propose does not require anaerobic conditions. Thus, we decided to test this new protocol to overcome one of the most important disadvantages of organosulfur compounds—their insufficient thermal and mechanical stability that was proved not to be up to the highest standards^30‚31^.

In order to achieve our goal, we set our focus on silsesquioxanes (SQ) since there have been no reports of β-sulfenylation of carbonyl compounds using this type of compounds. SQs can be defined as nanometric cage moieties with the general formula of [RSiO_1.5_]_n_. Their main characteristics are complete nontoxicity and robust, thermally stable siloxane framework ^32^ . SQs are also called hybrid compounds and due to their nature they can be easily adjusted to serve different applications^33–37^. Nowadays, they are mostly used in medicine as drug nanocarriers capable of binding with drug molecules both physically (using absorption^ 38‚39^) and chemically (creating chemical bonds ^40^) or as a significant component of various types of hydrogels used as wound band-aids^ 41–43^. Thanks to their properties, incorporatinion of SQ units guarantees a significant improvement in the stability of final products^44‚45^.

The aim of this study is to propose a new method of mercaptopropylisobutyl-POSS (**SQ-SH**) functionalization with a wide range of different unsaturated carbonyls, e.g., esters, ketones, ynals and demonstrate its effectiveness. The proposed protocol constitutes a highly effective tool for the efficient synthesis of new derivatives, while following the green chemistry principles. Another objective of our study was to assess the thermal properties before and after the incorporation of SQ unit to the bioactive compound, on the basis of TGA analysis that is commonly used with biomedical substances ^46‚47^.

## Results and discussion

### Design and optimization of the reaction system

**Fig. 1:**
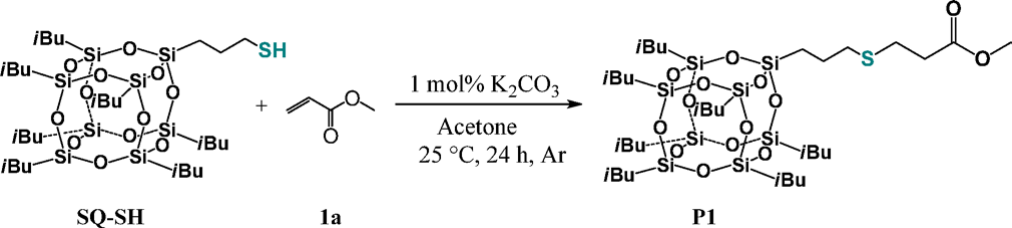
Hydrothiolation of mercaptopropylisobutyl POSS (**SQ-SH**) with methyl acrylate (**1a**).

Our examination commenced with the reaction between equimolar amounts of mercaptopropylisobutyl POSS (**SQ-SH**) and methyl acrylate (**1a**) using 1 mol% of potassium carbonate (K_2_CO_3_). The process was performed in 10 mL stainless steel jar with one stainless steel ball (φ = 7 mm) using a Retsch MM200 mixer working at 30 Hz. Unfortunately, despite using the same conditions as those described for simple thiols in our previous paper, we did not observe any transformation of reagents. An increase in the base loading to 10 mol % and a 36-fold increase in the reaction time also turned out to be ineffective. Therefore, we decided to check the possibility of **SQ-SH** functionalization using classical, non-mechanochemical method. Addition of the equimolar ratio of reagents to an acetone solution of 1 mol% of K_2_CO_3_ at 25 °C, resulted only in 30% conversion of the substrates after 24 h but ^1^H NMR analysis of the reaction mixture revealed selective formation of the expected product (**P1**) (Fig. 1):

This achievement prompted us to continue our studies and perform a wide range of optimization tests in order to select the most effective system permitting the increase in the yield of the final product **P1** in the mildest possible conditions. As shown in Table 1, temperature had a major impact on the outcome of the tested process. From among the temperatures tested, the optimal performance of the reaction was observed at 60 °C (Table 1, Entries 7–10). When the process was carried out at higher temperatures, no significant changes in the course of the reaction were noted (Table 1, Entry 12 vs 13). On the other hand, lowering the temperature below 60 °C resulted in reduced formation of the expected product **P1** (Table 1, Entries 3–6). Optimization tests indicated also that the concentration of the salt had influence on the reaction effectiveness. When the base loading was lower than 0.5 mol%, complete conversion of substrates was not achieved, even though the reaction time was extended to 72 h (Table 1, Entries 11 and 12) or the temperature was increased to 70 °C (Table 1, Entry 13). We have discovered also that the process did not require anaerobic conditions, besides, no oxidation leading to disulfides was observed (Table 1, Entry 4 vs 5). The experiments carried out in the absence of a base did not afford any products, even though the reaction time was extended to 96 h (See ESI, Table S1). Results gathered in Table 1 reveal that the outcome of the hydrothiolation process depends also on the reaction medium. From among all the green solvents tested, acetone was found to be the best one, although it was observed that the process could be efficiently carried out in ethanol and isopropanol, without a significant decrease in the yield of **P1** product (Table 1, Entries 16–17 and 10). An effective transformation of reagents was not observed when the process was carried out in MTBE and ethyl acetate, even after significantly extending the reaction time (Table 1, Entries 14–15). Conditions of entry 10 were found optimal as they allowed quantitative conversion of reagents by using a relatively low salt loading.

**Table 1:** Optimization of the reaction conditions.

Entry	T	K_2_CO_3_	Time	Aerobic conditions	Yield of P1^[e]^ [%]
	[°C]	[mol%]	[h]		
1	25	1	24	−	30
2	25	5	24	−	100
3	25	10	24	−	95
4	25	10	72	−	75
5	25	10	72	−	100
6	40	10	24	+	99
7	60	10	3	+	100
8	60	5	3	+	48
9	60	1	5	+	100
**10**	**60**	**0.5**	**5**	+	**98**
11	60	0.1	18	+	30
12	60	0.1	72	+	99
13	70	0.1	72	+	0
14^[a]^	60	0.5	24	+	75
15^[b]^	60	0.5	24	+	35
16^[c]^	60	0.5	5	+	96
17^[d]^	100	0.5	5	+	97

Reaction conditions: Acetone, [**SQ-SH**]:[**1a**]  = 1:1.

^[a]^MIBK was used instead of acetone.

^[b]^Ethyl acetate was used instead of acetone.

^[c]^Ethanol was used instead of acetone.

^[d]^Isopropanol was used instead of acetone.

^[e]^Determined by ^1^H NMR spectroscopy of the crude reaction mixture.

### Scope of the reaction

**Fig. 2:**
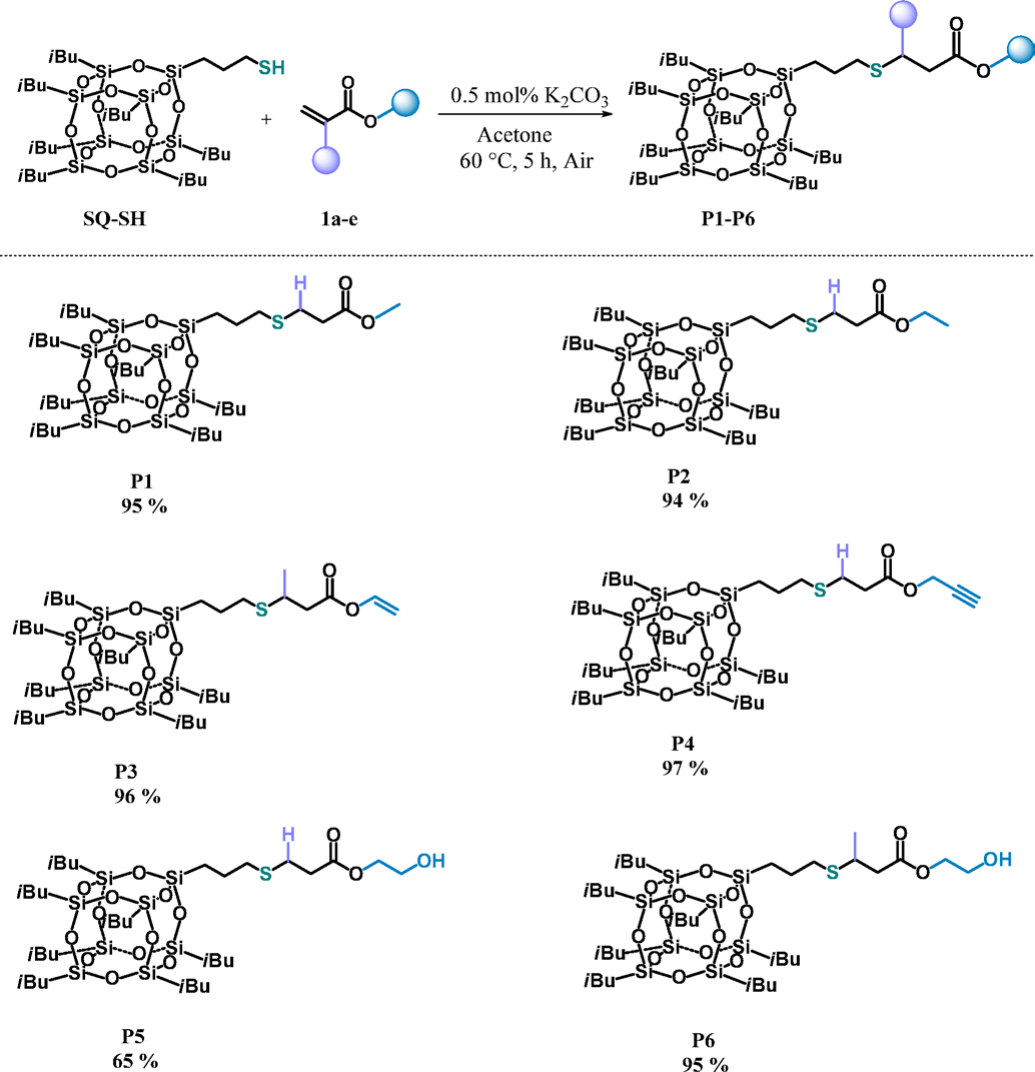
Functionalization of mercaptopropylisobutyl POSS (**SQ-SH**) with esters (**1a–e**) (experimental conditions: acetone, 60 °C, 5 h, (**SQ-SH**):(**1a–e**) = 1:1, [K_2_CO_3_] = 0.5 mol%, air). Isolated yields are given under the structure of each product.

Following our successful initial findings, we carefully explored the substrate scopes of the reactions under optimized conditions (Table 1, Entry 10). In the first series of experiments, we checked the reactivity of other commercially available esters containing ethyl (**1b**), vinyl (**1c**), propargyl (**1d**) and hydroxyl (**1e, 1f.**) groups (Fig. 2). For all substrates tested, we achieved quantitative yields and complete chemoselectivity towards SMA products formation. No significant differences in the reaction course were observed, which proves that for the synthetic protocol works with different moieties. What is important, the esters having unsaturated bonds (**1c**, **1d**) or hydroxyl group (**1e**, **1f.**) in their structures were also applied in the reactions with **SQ-SH** and then the reactions led to new products susceptible to further modifications (**P3**-**P6**). Interestingly, that materials **P5** and **P6** have gained solubility in methanol, which extends the possibility of their isolation by precipitation methods. All products (**P1**-**P6**) were isolated and characterized by spectroscopic and spectrometric methods (See ESI).

**Fig. 3:**
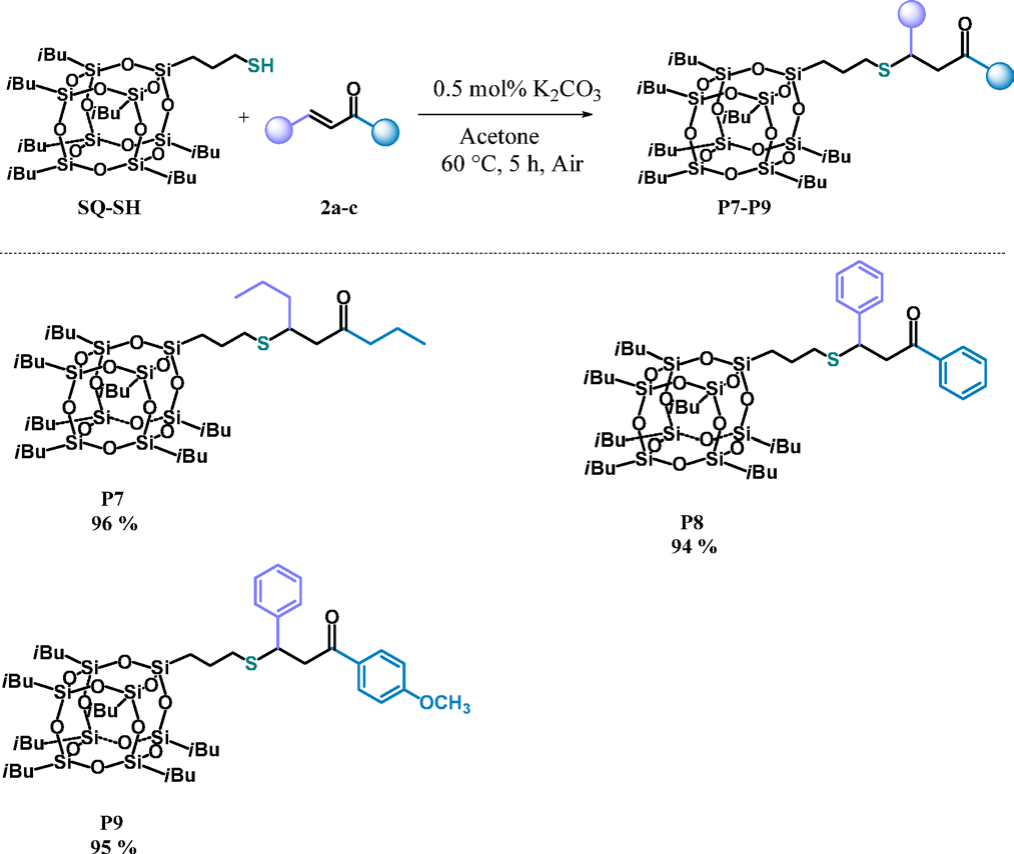
Functionalization of mercaptopropylisobutyl POSS (**SQ-SH**) with ketones (**2a–c**) (experimental conditions: acetone, 60 °C, 5 h, (**SQ-SH**): (**1a–e**) = 1:1, [K_2_CO_3_] = 0.5 mol%, air). Isolated yields are given under the structure of each product.

To investigate the scope of the proposed reaction with respect to the carbonyl reagent type, α,β-unsaturated ketones were used instead of esters (Fig. 3). Thus, **SQ-SH** was reacted with alkyl ketone (**2a**) and two commercially available chalcones (**2b**, **2c**), that are very important compounds because of their strong medical properties leading to a vast number of applications^4^ .

To our great satisfaction, the reactions led to the expected products (**P7**-**P9**), irrespective of the nature of the carbonyl compounds used. We did not observe the formation of other products of competitive reactions for any of the reagents studied. All materials were isolated with high yields, which proved that both alkyl and aryl ketones can be successfully adapted to the presented protocol.

**Fig. 4:**
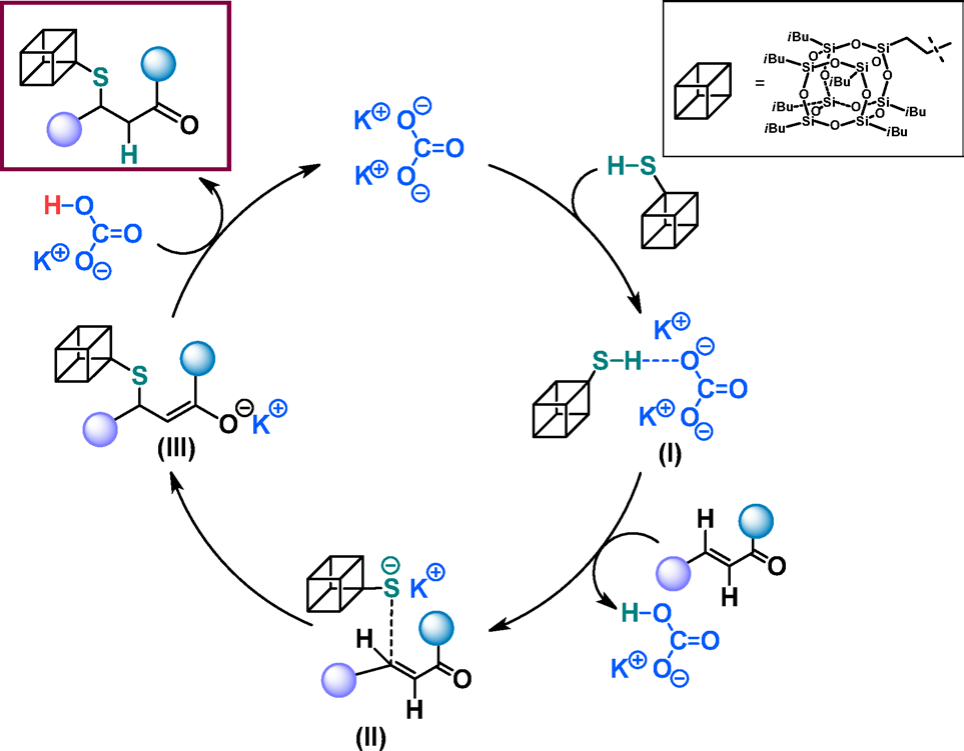
Proposed mechanism for the formation of **P1–P9**.

Based on literature and our previous research on thioester synthesis from α,β-unsaturated aldehydes with thiols^29,48–51^, we proposed a mechanism of the reaction depicted in Fig. 4.

The products **P1**-**P9** are obtained as a result of sulfa-Michael addition according to the proton transfer mechanism^ 48–51^. Potassium carbonate functions as a base which deprotonates the acidic thiol molecule to form K_2_CO_3_-thioxy intermediate (**I**), which then reacts with ketone or ester in the conjugate addition ^52^  (**II**). Protonation of the resulting intermediate (**III**) gives SMA product and regenerates the catalyst.

**Fig. 5:**
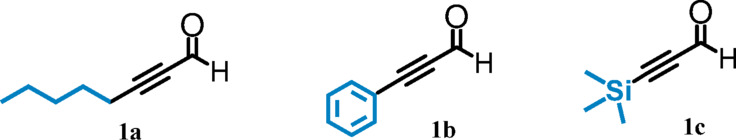
Structures of ynals tested.

In the next step, we applied the proposed procedure to unsaturated carbonyl compounds containing not double, but triple carbon–carbon bonds in the direct neighborhood of carbonyl group. As presented in Fig. 5, we tested the reactivity of alkyl (**3a**), aryl (**3b**) and silyl (**3c**) propargyl aldehydes.

**Fig. 6:**
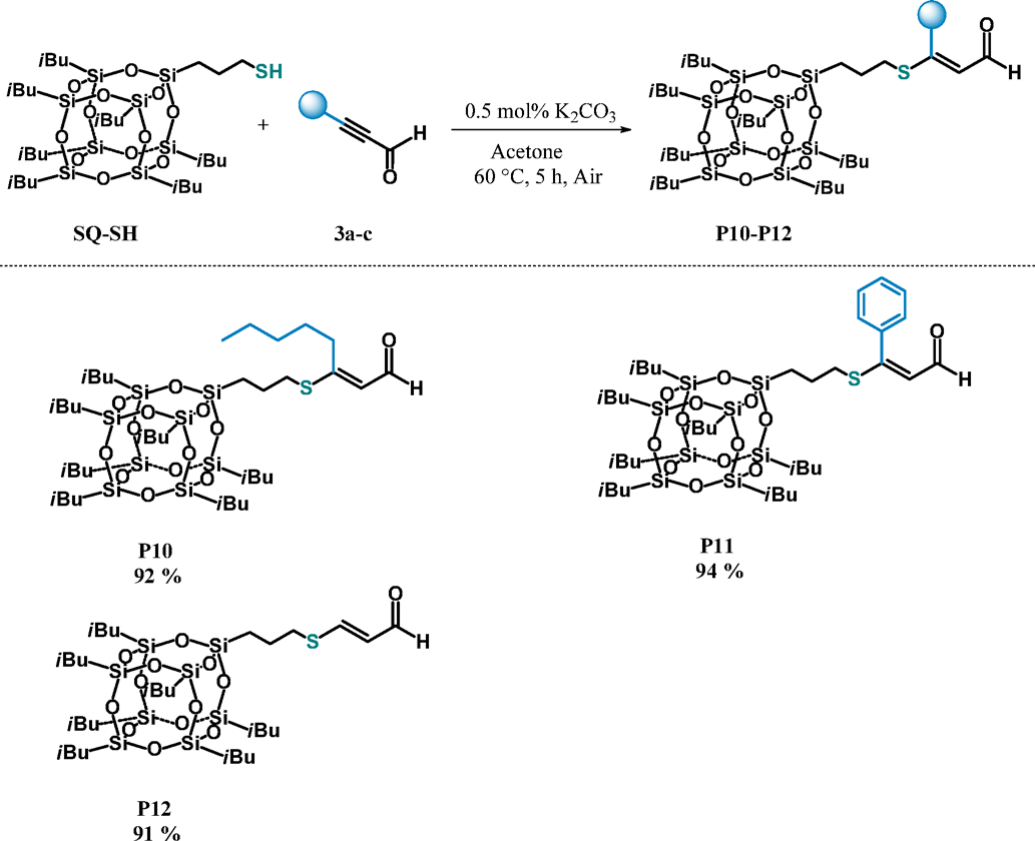
Functionalization of mercaptopropylisobutyl POSS (**SQ-SH**) with ynals (**3a–c**) (experimental conditions: acetone, 60 °C, 5 h, (**SQ-SH**):(**3a–e) = **1:1, [K_2_CO_3_] = 0.5 mol%, air). Isolated yields are given under the structure of each product.

We turned our attention to functionalization of this kind of compounds, because they can be used as versatile synthons in the synthesis of various heterocycles (e.g. indolizines) ^53^. Moreover, they are the substrates more demanding for functionalization than enals because their hydrothiolation might generate β-*E* and β-*Z* isomers, so it is advisable to expand the knowledge of their modifications. Therefore, we probed the reactivity of selected ynals (**1a-c**) toward **SQ-SH** (Fig. 6).

The results presented in Fig. 6 show that irrespective of the type of acetylenes and silanes used, all reactions are stereoselective and lead exclusively to the product of *E* geometry of the resulting substituted vinylene bonds. During the reaction with 3-(trimethylsilyl)prop-2-ynal (**3c**) we observed the cleavage of the TMS group that is consistent with the results of our previous research ^53^ indicating that in such circumstances TMS elimination can be catalyzed by the presence of a base that is also used in our protocol. We have also made multiple attempts at obtaining (bis)substituted product, however, due to the huge steric hindrance caused by the silsesquioxane molecule such a reaction did not occur. An attempt at using a smaller thiol (benzyl mercaptan) as the alternative to the second eq. of SQ, has also failed.

**Fig. 7:**
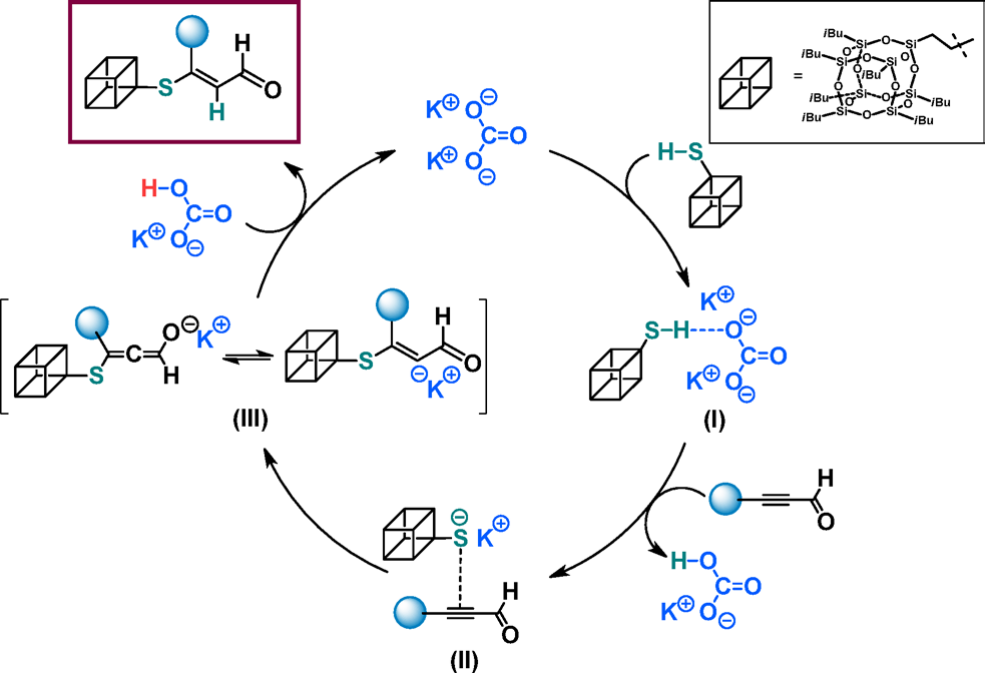
Proposed mechanism for the formation of** P10–P12**.

Similarly to the assumed mechanism of saturated products formation, we proposed a mechanism of creating of unsaturated SMA adducts based on available literature and our previous research on ynals functionalization ^53^ (Fig. 7).

The whole mechanism commences from the reaction between K_2_CO_3_ and **SQ-SH** leading to form NHC-thioxy intermediate (**I**). In the next step, the electron withdrawing carbonyl group activates the ynal molecule ^54^ which is a perfect substrate for immediate conjugate addition with the obtained intermediate (**I**). This reaction lead to intermediate (**III**), which can remain in equilibrium with allenic enolate ^55^. Such a species is highly reactive and immediately leads to formation of *E*-products and regenerated salt.

### The preparative scale of synthesis of product P1

**Fig. 8:**
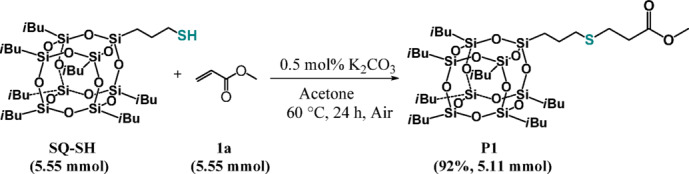
Scaled-up synthesis of **P1**.

To demonstrate the synthetic utility of the designed protocol, a gramscale hydrothiolation of **SQ-SH** with **1a** was performed (Fig. 8). The result obtained makes it clear that the proposed methodology has a significant application potential.

Within this study, twelve products were obtained, isolated and characterized by spectroscopic methods (See ESI for details). These materials are air-stable which makes them very attractive for further applications. The presented derivatives are new compounds that have never been published before.

### Thermal properties

**Fig. 9:**
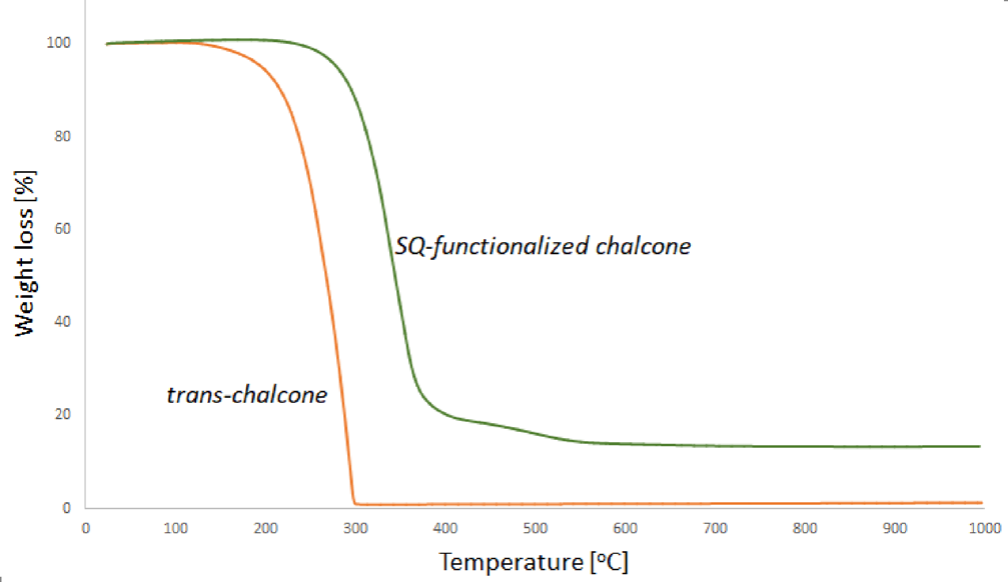
TGA curves (N_2_) of pure trans-chalcone (**2b**) and SQ-functionalized chalcone (**P8**).

The thermal stability of selected *trans*-chalcone (**2b**) and SQ-based products (**P8**) was investigated with thermogravimetric analysis in a nitrogen atmosphere. The results are summarized in Table 2 and presented in Fig. 9. Analysis of the obtained thermograms recorded in N_2_ atmosphere revealed that the incorporation of silsesquioxane moiety into the chalcone unit (**2a**) strongly influenced the thermal properties of the resulting product (**P8**). The 5% mass loss of the substrate was observed at 196 °C, meanwhile the initial decomposition temperature of the product increased to 279 °C. This is strictly related to the high stability of the SQ core, because of the high dissociation energy of the Si–O bond, which is much higher than for typical organic units containing C–C and C–H bonds^56‚57^. Moreover, the final transition of for *trans*-chalcone is completed below 300 °C, resulting in the total weight loss (100%). No total weight loss was observed in the SQ-functionalized chalcone, even at 1000 °C.

**Table 2:** Thermal properties of pure *trans*-chalcone (**2b**) and SQ-based chalcone (**P8**).

Entry	Compound	Temp. of mass lost [°C]		Residue [%]	
		5%	50%	300 °C	1000 °C
1	*trans*-chalcone	196	267	0	0
2	SQ-chalcone	279	344	88	13

Measurements made in N_2_ atmosphere.

## Conclusion

To sum up, a new practical protocol for the efficient synthesis of a novel class of functionalized SQs, based on the processes of 1,4-addition of **SQ-SH** to ketone, esters as well as ynals, catalyzed by potassium carbonate, was designed and optimized. For all combinations of the reagents tested, the reactions proceeded effectively leading to exclusive formation of the expected materials. The most significant advantages of the newly developed methodology include no need of transition metals use, low concentrations of organocatalyst, the use of a green solvent (acetone), elimination of any other additives and full chemoselectivity. Additionally, all syntheses can be performed in aerobic conditions in a relatively short reaction time, which is fully consistent with the rules of green chemistry^58^. The reactions are efficient and easily scalable to gram quantities, offering substantial benefits for practical applications. Finally, twelve new carbonyl-modified SQ-based materials were obtained and characterized by spectroscopic and spectrometric methods. The thermal analysis conducted for pure *trans*-chalcone and chalcone-SQ system has proved that implementation of silsesquioxyl moieties into the structure of carbonyl compound has a significant impact on thermal properties of the final materials.

## Methods

### General methods and reagents

Unless otherwise indicated, all operations were carried out under aerobic conditions. ^1^H NMR and ^13^C NMR spectra were recorded at 25 °C in CDCl_3_ on a Varian 400 operating at 402.6 and 101.2 MHz, respectively. ^29^Si NMR were recorded on a Brucker Ascend 400 Nanobay operating at 79.50 MHz. Chemical shifts are reported in ppm with reference to the residual solvent peaks for ^1^H and ^13^C NMR and to TMS for ^29^Si NMR. Thin layer chromatography (TLC) was conducted on plates coated with a 250 μm thick silica gel layer and column chromatography was performed on silica gel 60 (70–230 mesh). ESI–MS spectra were obtained using Synapt Gs-S HDMS (Waters) mass spectrometer with electrospray ion source and quadrupole-time-of-flight analyzer with resolving power of FWMH 38,000. Acetonitrile was used as the sample solvent. The Capillary Voltage was set to 4.5 kV, the sampling was set to 40 and the source temperature was equal to 120 °C. The most abundant ions in the ESI–MS spectra were sodiated and potassiated ions of desired products. The TGA analyses were performed using a thermogravimetric analyzer TGA4000. The heating rate was set to 10 °C/min. The analyses were made using nitrogen as carrying gas at the flow rate of 20 ml per minute. The samples (approx. 10 mg) were heated from 0 to 1000 °C. The high-resolution mass spectra (HRMS) were recorded on an Impact HD mass spectrometer (Brucker) equipped with an electrospray ion source and quadrupole-time-of-flight analyzer with resolving power of FWMH 20,000. The main signal in all spectra corresponds to the sodiated ions (M + Na^+^) of expected products. Fourier Transform-Infrared (FT-IR) measurements were performed by using a Thermo Fischer Nicolet iS50 spectrometer (Thermo Scientific). In all cases, 256 scans in the range of 4000–400 cm^−1^ were collected with 2 cm^−1^ resolution. The solid samples were measured using standard ATR technique. The obtained spectra were subjected to baseline correction and normalization.

All reagents were commercially available and used without further purification. The chemicals were purchased from the following sources: mercaptopropylisobutyl-POSS (Hybrid Plastic), ketones, esters and ynals (Chemat), potassium carbonate (Merck), chloroform-d_1_ (Deutero) and acetone, DCM, *n*-hexane (Fisher Chemical).

### General procedure for catalytic tests

An oven-dried 5 mL glass reactor equipped with a magnetic stirring bar was charged in aerobic conditions with K_2_CO_3_ (0.06 mg, 4.20 × 10^–7^ mol), **SQ-SH** (75.00 mg, 8.41 × 10^–5^ mol), α,β-unsaturated carbonyl compound (8.41 × 10^–5^ mol) and acetone (1 mL). The reaction mixture was stirred at 60 °C for 5 hours. After that, the solvent was evaporated under vacuum and the residue was analyzed using ^1^H NMR spectroscopy.

### General procedure for the synthesis of products P1-P12

An oven-dried 5 mL glass reactor equipped with a magnetic stirring bar was charged in aerobic conditions with K_2_CO_3_ (0.12 mg, 8.7 × 10^–6^ mol), **SQ-SH** (150.00 mg, 1.68 × 10^–4^ mol), α,β-unsaturated carbonyl compound (1.68 × 10^–4^ mol) and acetone (1 mL). The reaction mixture was stirred at 60 °C for 5 h. After that, the solvent was evaporated under vacuum and the residue was purified by column chromatography on silica gel using *n*-hexane or 2:1 v/v mixture of *n*-hexane and DCM as eluents. The evaporation of the solvents afforded analytically pure compounds.

### Synthesis of product P1 on a preparative scale

An oven-dried 15 mL glass reactor equipped with a magnetic stirring bar was charged with acetone (2 mL), **SQ-SH** (1.5 g, 1.68 × 10^–3^ mol), methyl acrylate (**1a**) (151.50 µL, 1.68 × 10^–3^ mol) and K_2_CO_3_ (1.16 mg, 8.40 × 10^–6^ mol). The reaction mixture was stirred at 60 °C for 24 h. After that, the solvent was evaporated under vacuum and the residue was purified by column chromatography on silica gel using *n*-hexane or 2:1 v/v mixture of *n*-hexane and DCM as eluents. The evaporation of the solvents afforded analytically pure compound **P1**.

## Supplementary Information

Below is the link to the electronic supplementary material.

Supplementary Material 1

## Data Availability

All data generated or analyzed during this study are included in this published article and its supplementary information file.
