# Supplementary material for: Synthesis of carbonyl-functionalized mercaptosilsesquioxane
Source: Sci Rep. 2025 Aug 11;15:29286. doi: 10.1038/s41598-025-14360-x (PMC12336308; doi:10.1038/s41598-025-14360-x)
Supplement: Supplementary file 1 — Supplementary Material 1 [file 41598_2025_14360_MOESM1_ESM.pdf]

Supporting Information

For

Synthesis of carbonyl-functionalized mercaptosilsesquioxane

Kamil Hanek and Patrycja Żak[a,*]

[a] *Department of Organometallic Chemistry, Faculty of Chemistry, Adam Mickiewicz University in Poznan, Uniwersytetu Poznańskiego 8, 61-614 Poznań, Poland. E-mail: pkw@amu.edu.pl*

CONTENTS:

1. **Analytical data of products P1-P12** **S-2**
2. **FT-IR spectra of products P1, P9 and P12** **S-6**
3. **NMR spectra of products P1-P12** **S-7**

1. Analytical data of products P1-P12

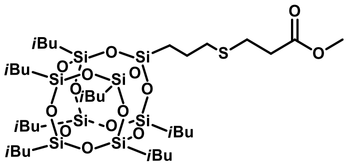<br>**Product P1**	Yellow solid, isolated yield: 95%; ^{1}H NMR (400 MHz, $CDCl_3$, 296K): δ (ppm) 0.56 – 0.64 (m, 14H, CH_2), 0.68 – 0.73 (m, 2H, CH_2), 0.92 – 0.99 (m, 42H, CH_3), 1.64 – 1.71 (m, 2H, CH_2), 1.80 – 1.89 (m, 7H, CH), 2.51 – 2.56 (m, 2H, CH_2), 2.60 (t, 2H, J_{HH} = 7.3 Hz, SCH_2), 2.76 (t, 2H, J_{HH} = 7.3 Hz, SCH_2), 3.70 (s, 3H, OCH_3); ^{13}C NMR (100 MHz, $CDCl_3$, 296K): δ (ppm) 11.59 (CH_2), 22.45 (CH_2), 22.49 (CH_2), 23.02 (CH_2), 23.83 (CH_2), 23.84 (CH), 23.88 (CH), 25.65 (CH_3), 25.68 (CH_3), 26.72 (CH_2), 34.72 (CH_2), 34.93 (SCH_2), 51.70 (OCH_3), 172.33 (CO); ^{29}Si NMR (100 MHz, $CDCl_3$, 296K): δ (ppm) -67.71, -67.94, -68.02; MS (ESI+): m/z 999 $[M+Na]^+$; HRMS (ESI+): calcd. for $C_{35}H_{76}NaO_{14}SSi_8$: *m/z* 999.3008; found: 999.3008; FT-IR (ATR, cm^{-1}): 3000 ν(C-H), 1740 ν(C=O), 1450, 1380 asymmetric and symmetric δ(CH_2), 1200 ν(C-O), 1100 ν(Si -O), 1050 δ(C-O), 870 δ(Si-O), 750 ν(Si-C) and ν(S-C), 550 δ(Si-O) and δ(S-C).
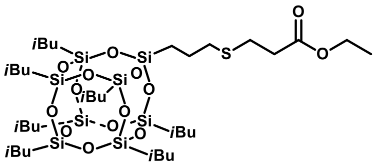<br>**Product P2**	White solid, isolated yield: 94%; ^{1}H NMR (400 MHz, $CDCl_3$, 296K): δ (ppm) 0.54 – 0.64 (m, 14H, CH_2), 0.68 – 0.73 (m, 2H, CH_2), 0.90 – 1.02 (m, 42H, CH_3), 1.26 (t, 3H, J_{HH} = 7.1 Hz, CH_3CH_2), 1.65 – 1.72 (m, 2H, CH_2), 1.80 – 1.90 (m, 7H, CH), 2.51 – 2.60 (m, 4H, CH_2), 2.76 (t, 2H, J_{HH} = 7.4 Hz, SCH_2), 4.15 (q, 2H, J_{HH} = 7.1 Hz, CH_3CH_2); ^{13}C NMR (100 MHz, $CDCl_3$, 296K): δ (ppm) 11.60 (CH_2), 14.19 (CH_3), 22.46 (CH_2), 22.50 (CH_2), 23.05 (CH_2), 23.83 (CH), 23.87 (CH), 25.64 (CH_3), 25.67 (CH_3), 26.80 (CH_2), 34.97 (CH_2), 34.99 (CH_2), 60.56 (OCH_2), 171.88 (CO); ^{29}Si NMR (100 MHz, $CDCl_3$, 296K): δ (ppm) -67.65, -67.89, -67.95; MS (ESI+): m/z 1013 $[M+Na]^+$; HRMS (ESI+): calcd. for $C_{36}H_{78}NaO_{14}SSi_8$: m/z 1013.3164; found: 1013.3161.
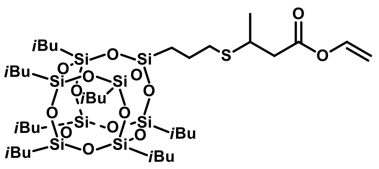<br>**Product P3**	White solid, isolated yield: 96%; ^{1}H NMR (400 MHz, $CDCl_3$, 296K): δ (ppm) 0.53 – 0.66 (m, 14H, CH_2), 0.67 – 0.73 (m, 2H, CH_2), 0.82 – 1.11 (m, 42H, CH_3), 1.30 (d, 3H, J_{HH} = 6.9 Hz, CH_3), 1.64 – 1.72 (m, 2H, CH_2), 1.79 – 1.91 (m, 7H, CH), 2.48 – 2.56 (m, 2H, CH_2), 2.57 – 2.62 (m, 1H, CH), 2.69 – 2.76 (m, 1H, CH_2), 2.82 – 2.87 (m, 1H, CH_2), 4.60 (dd, 1H, J_{HH} = 6.3, 1.7 Hz, $HC=CH_2$), 4.91 (dd, 1H, J_{HH} = 14.0, 1.7 Hz, $HC=CH_2$), 7.29 (dd, 1H, J_{HH} = 14.0, 6.3 Hz, $HC=CH_2$); ^{13}C NMR (100 MHz, $CDCl_3$, 296K): δ (ppm) 11.57 (CH_2), 22.45 (CH_3), 22.48 (CH_2), 23.06 (CH_2), 23.84 (CH), 23.88 (CH), 25.68 (CH_3), 34.89 (CH), 35.48 (CH_2), 40.12 (CH_2), 97.90, 141.20, 172.20 (CO); ^{29}Si NMR (100 MHz, $CDCl_3$, 296K): δ (ppm) -67.65, -67.88, -67.95; MS (ESI+): m/z 1025 $[M+Na]^+$; HRMS (ESI+): calcd. for $C_{37}H_{78}NaO_{14}SSi_8$: m/z 1025.3164; found: 1025.3169.

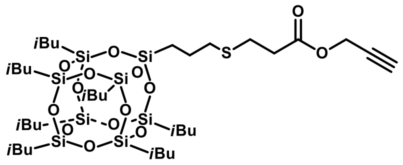 **Product P4**	White solid, isolated yield: 97%; ^{1}H NMR (400 MHz, $CDCl_3$, 296K): δ (ppm) 0.56 – 0.63 (m, 14H, CH_2), 0.67 – 0.74 (m, 2H, CH_2), 0.93 – 0.97 (m, 42H, CH_3), 1.63 – 1.72 (m, 2H, CH_2), 1.80 – 1.88 (m, 7H, C*H*), 2.47 (t, 1H, J_{HH} = 2.5 Hz, ≡C*H*), 2.52 – 2.56 (m, 2H, CH_2), 2.63 – 2.67 (m, 2H, CH_2), 2.75 – 2.79 (m, 2H, CH_2), 4.70 (d, 2H, J_{HH} = 2.5 Hz, SCH_2); ^{13}C NMR (100 MHz, $CDCl_3$, 296K): δ (ppm) 11.57 (*C*H_2), 22.43 (*C*H_2), 22.46 (*C*H_2), 22.98 (*C*H_2), 23.83 (*C*H_2), 23.87 (*C*H), 25.66 (*C*H_3), 25.68 (*C*H_3), 26.48 (*C*H_2), 34.65 (*C*H_2), 34.93 (*C*H_2), 74.97 (≡*C*H), 77.45 (≡*C*), 171.07 (*C*O); ^{29}Si NMR (100 MHz, $CDCl_3$, 296K): δ (ppm) -67.65, -67.88, -67.99; MS (ESI+): m/z 1023 $[M+Na]^+$; HRMS (ESI+): calcd. for $C_{37}H_{76}NaO_{14}SSi_8$: m/z 1023.3008; found: 1023.3001.
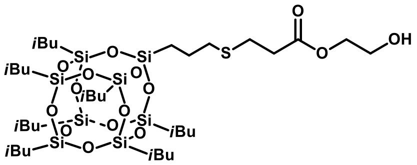 **Product P5**	White solid, isolated yield: 95%; ^{1}H NMR (400 MHz, $CDCl_3$, 296K): δ (ppm) 0.56 – 0.63 (m, 14H, CH_2), 0.68 – 0.73 (m, 2H, CH_2), 0.91 – 0.99 (m, 42H, CH_3), 1.64-1.71 (m, 2H, CH_2), 1.80 – 1.88 (m, 7H, C*H*), 1.96 (br s, 1H, O*H*), 2.52 – 2.58 (m, 2H, CH_2), 2.62 – 2.68 (m, 2H, CH_2), 2.75 – 2.81 (m, 2H, SCH_2), 3.81 – 3.85 (m, 2H, OCH_2), 4.23 – 4.27 (m, 2H, OCH_2); ^{13}C NMR (100 MHz, $CDCl_3$, 296K): δ (ppm) 11.61 (*C*H_2), 22.48 (*C*H_2), 22.52 (*C*H_2), 23.02 (*C*H_2), 23.84 (*C*H), 23.89 (*C*H), 25.66 (*C*H_3), 25.67 (*C*H_3), 26.87 (*C*H_2), 34.72 (*C*H_2), 34.95 (S*C*H_2), 61.14 (O*C*H_2), 66.24 (O*C*H_2), 172.06 (*C*O); ^{29}Si NMR (100 MHz, $CDCl_3$, 296K): δ (ppm) -67.63, -67.88, -68.01; MS (ESI+): m/z 1029 $[M+Na]^+$; HRMS (ESI+): calcd. for $C_{36}H_{78}NaO_{15}SSi_8$: m/z 1029.3113; found: 1029.3039.
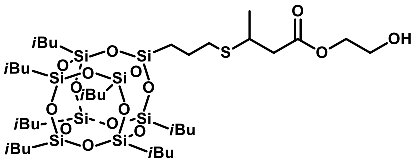 **Product P6**	White solid, isolated yield: 97%; ^{1}H NMR (400 MHz, $CDCl_3$, 296K): δ (ppm) 0.54 – 0.66 (m, 14H, CH_2), 0.67 – 0.72 (m, 2H, CH_2), 0.84 – 1.04 (m, 42H, CH_3), 1.27 (d, 3H, J_{HH} = 6.9 Hz, CH_3), 1.64-1.71 (m, 2H, CH_2), 1.80 – 1.91 (m, 7H, C*H*), 2.05 (br s, 1H, O*H*), 2.51 – 2.56 (m, 2H, CH_2), 2.60 – 2.64 (m, 1H, C*H*), 2.72 – 2.80 (m, 2H, SCH_2), 3.80 – 3.85 (m, 2H, OCH_2), 4.20 – 4.25 (m, 1H, OCH_2), 4.28 – 4.33 (m, 1H, OCH_2); ^{13}C NMR (100 MHz, $CDCl_3$, 296K): δ (ppm) 16.60 (*C*H_2), 16.97 (*C*H_3), 22.47 (*C*H_2), 22.51 (*C*H_2), 23.05 (*C*H_2), 23.83 (*C*H), 23.88 (*C*H), 25.65 (*C*H_3), 25.67 (*C*H_3), 25.43 (*C*H_2), 40.19 (S*C*H_2), 61.14 (O*C*H_2), 66.19 (O*C*H_2), 175.24 (*C*O); ^{29}Si NMR (100 MHz, $CDCl_3$, 296K): δ (ppm) -67.64, -67.87, -68.03; MS (ESI+): m/z 1043 $[M+Na]^+$; HRMS (ESI+): calcd. for $C_{37}H_{80}NaO_{15}SSi_8$: m/z 1043.3270; found: 1043.3252.

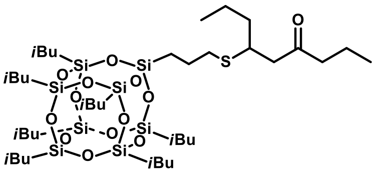 **<u>Product P7</u>**	White solid, isolated yield: 96%; ^{1}H NMR (400 MHz, $CDCl_3$, 296K): δ (ppm) 0.53 – 0.64 (m, 14H, CH_2), 0.67 – 0.72 (m, 2H, CH_2), 0.88 (t, 3H, J_{HH} = 7.0 Hz, CH_3), 0.91 – 0.96 (m, 42H, CH_3), 1.22-1.33 (m, 4H, CH_2), 1.36 – 1.44 (m, 2H, CH_2), 1.47-1.54 (m, 2H, CH_2), 1.62 – 1.68 (m, 2H, CH_2), 1.79 – 1.90 (m, 7H, CH), 2.17 (s, 3H, CH_3), 2.47 – 2.54 (m, 2H, CH_2), 2.61 – 2.71 (m, 2H, CH_3CH_2), 3.04 – 3.10 (m, 2H, CH); ^{13}C NMR (100 MHz, $CDCl_3$, 296K): δ (ppm) 11.85 (CH$_2$), 14.02 (CH$_3$), 22.43 (CH$_2$), 22.47 (CH$_2$), 22.55 (CH$_3$), 23.39 (CH$_2$), 23.83 (CH), 23.87 (CH), 25.67 (CH$_3$), 25.68 (CH$_3$), 26.44 (CH$_2$), 30.76 (CH$_2$), 31.64 (CH$_2$), 33.78 (CH$_2$), 35.23 (CH), 40.60 (SCH$_2$), 49.76 (OCH$_2$), 206.94 (CO); ^{29}Si NMR (100 MHz, $CDCl_3$, 296K): δ (ppm) -67.66, -67.89, -67.97; MS (ESI+): m/z 1053 $[M+Na]^+$; HRMS (ESI+): calcd. for $C_{40}H_{86}NaO_{13}SSi_8$: m/z 1053.3841; found: 1053.3823.
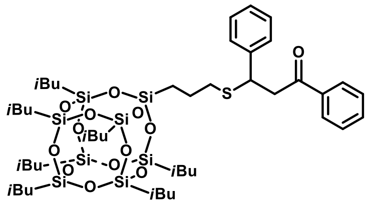 **<u>Product P8</u>**	White solid, isolated yield: 94%; ^{1}H NMR (400 MHz, $CDCl_3$, 296K): δ (ppm) 0.56– 0.65 (m, 16H, CH_2), 0.94 – 0.98 (m, 42H, CH_3), 1.56 – 1.66 (m, 2H, CH_2), 1.81 – 1.88 (m, 7H, CH), 2.28 – 2.41 (m, 4H, CH_2), 3.47 – 3.60 (m, 2H, SCH_2), 4.53 – 4.60 (m, 1H, SCH), 7.19 – 7.23 (m, 1H, C_6H_5), 7.28 – 7.32 (m, 2H, C_6H_5), 7.40 – 7.45 (m, 4H, C_6H_5), 7.52 – 7.56 (m, 1H, C_6H_5), 7.90 – 7.92 (m, 2H, C_6H_5); ^{13}C NMR (100 MHz, $CDCl_3$, 296K): δ (ppm) 11.78 (CH$_2$), 22.45 (CH$_2$), 22.52 (CH$_2$), 22.78 (CH$_2$), 23.84 (CH), 23.88 (CH), 25.67 (CH$_3$), 25.69 (CH$_3$), 34.35 (CH$_2$), 44.17 (SCH$_2$), 45.54 (CH), 127.15, 127.82, 128.06, 128.47, 128.56, 133.11, 136.89, 142.21, 196.82 (CO); ^{29}Si NMR (100 MHz, $CDCl_3$, 296K): δ (ppm) -67.72, -67.96, -68.10; MS (ESI+): m/z 1121 $[M+Na]^+$; HRMS (ESI+): calcd. for $C_{46}H_{82}NaO_{13}SSi_8$: m/z 1121.3528; found: 1121.3529.
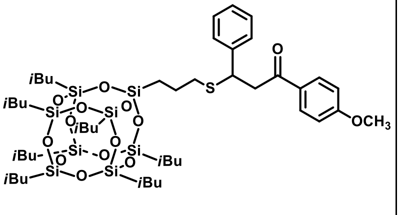 **<u>Product P9</u>**	White solid, isolated yield: 95%; ^{1}H NMR (400 MHz, $CDCl_3$, 296K): δ (ppm) 0.57 – 0.62 (m, 16H, CH_2), 0.94 – 0.97 (m, 42H, CH_3), 1.60 – 1.66 (m, 2H, CH_2), 1.82 – 1.89 (m, 7H, CH), 2.28 – 2.37 (m, 2H, CH_2), 3.46 – 3.54 (m, 2H, SCH_2), 3.77 (s, 3H, OCH_3), 4.49 – 4.53 (m, 1H, SCH), 6.82 (d, 2H, J_{HH} = 8.7 Hz, C_6H_5), 7.32 (d, 2H, J_{HH} = 8.7 Hz, C_6H_5), 7.41 – 7.45 (m, 2H, C_6H_5), 7.52 – 7.56 (m, 1H, C_6H_5), 7.89 – 7.92 (m, 2H, C_6H_5); ^{13}C NMR (100 MHz, $CDCl_3$, 296K): δ (ppm) 11.79 (CH$_2$), 22.42 (CH$_2$), 22.48 (CH$_2$), 22.72 (CH$_2$), 23.83 (CH), 23.87 (CH), 25.67 (CH$_3$), 25.68 (CH$_3$), 34.24 (CH$_2$), 44.47 (SCH$_2$), 45.63 (CH), 55.18 (OCH$_3$), 113.81, 128.05, 128.84, 133.11, 134.04, 136.85, 158.56, 196.97 (CO); ^{29}Si NMR (100 MHz, $CDCl_3$, 296K): δ (ppm) -67.66, -67.90, -68.02; MS (ESI+): m/z 1151 $[M+Na]^+$; HRMS (ESI+): calcd. for $C_{47}H_{84}NaO_{14}SSi_8$: m/z 1151.3634; found: 1151.3632; FT-IR (ATR, cm^{-1}): 3050 ν(Ar-H), 3000 ν(C-H), 1715 ν(C=O), 1600-1400 ν(C=C), 1450, 1380 asymmetric and symmetric

	δ(CH_2), 1200 ν(C-O), 1100 ν(Si -O), 1050 δ(C-O), 870 δ(Si-O), 750 ν(Si-C) and ν(S-C), 600 γ(Ar-H), 550 δ(Si-O) and δ(S-C).
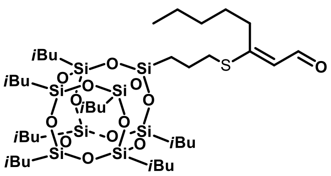 **Product P10**	Yellow solid, isolated yield: 92%; ^{1}H NMR (400 MHz, $CDCl_3$, 296K): δ (ppm) 0.58 – 0.62 (m, 14H, CH_2), 0.72 – 0.76 (m, 2H, CH_2), 0.88 - 0.92 (m, 3H, CH_3), 0.94 – 0.96 (m, 42H, CH_3), 1.32-1.38 (m, 4H, CH_2), 1.65-1.70 (m, 2H, CH_2), 1.78 – 1.90 (m, 9H, CH_2 and C*H*), 2.67 – 2.73 (m, 2H, CH_2), 2.79 (t, 2H, J_{HH} = 7.2 Hz, SCH_2), 5.78 (d, 1H, J_{HH} = 7.9 Hz, C*H*), 9.82 (d, 1H, J_{HH} = 7.9 Hz, C*H*); ^{13}C NMR (100 MHz, $CDCl_3$, 296K): δ (ppm) 11.83 (CH_2), 13.81 (CH_3), 21.31 (CH_2), 22.29 (CH_2), 22.43 (CH_2), 22.49 (CH_2), 23.83 (*C*H), 23.89 (*C*H), 25.66 (CH_3), 25.68 (CH_3), 31.13 (CH_2), 31.42 (CH_2), 33.45 (CH_2), 34.14 (SCH_2), 120.31, 169.88, 186.43 (*C*O); ^{29}Si NMR (100 MHz, $CDCl_3$, 296K): δ (ppm) -67.68, -67.93, -68.02; MS (ESI+): m/z 1037 $[M+Na]^+$; HRMS (ESI+): calcd. for $C_{39}H_{82}NaO_{13}SSi_8$: m/z 1037.3528; found: 1037.3519.
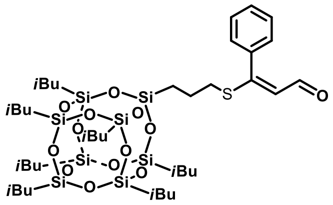 **Product P11**	Yellow solid, isolated yield: 94%; ^{1}H NMR (400 MHz, $CDCl_3$, 296K): δ (ppm) 0.58 – 0.62 (m, 14H, CH_2), 0.73 – 0.82 (m, 2H, CH_2), 0.94 – 0.96 (m, 42H, CH_3), 1.81 – 1.88 (m, 9H, CH_2 and C*H*), 2.89 (t, 2H, J_{HH} = 7.2 Hz, SCH_2), 6.07 (d, 1H, J_{HH} = 7.9 Hz, C*H*), 7.41-7.44 (m, 5H, C_6H_5), 9.27 (d, 1H, J_{HH} = 7.9 Hz, C*H*); ^{13}C NMR (100 MHz, $CDCl_3$, 296K): δ (ppm) 11.84 (CH_2), 21.54 (CH_2), 22.45 (CH_2), 22.50 (CH_2), 23.84 (*C*H), 23.90 (*C*H), 25.67 (CH_3), 25.68 (CH_3), 35.39 (SCH_2), 122.52, 128.48, 129.38, 130.08, 135.57, 167.78, 189.54 (*C*O); ^{29}Si NMR (100 MHz, $CDCl_3$, 296K): δ (ppm) -67.56, -67.85, -68.63; MS (ESI+): m/z 1059 $[M+K]^+$; HRMS (ESI+): calcd. for $C_{40}H_{76}NaO_{13}SSi_8$: m/z 1043.3058; found: 1043.3047.
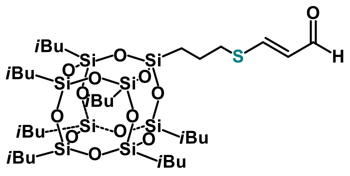 **Product P12**	Yellow solid, isolated yield: 91%; ^{1}H NMR (400 MHz, $CDCl_3$, 296K): δ (ppm) 0.56 – 0.65 (m, 14H, CH_2), 0.72 – 0.78 (m, 2H, CH_2), 0.90 - 1.01 (m, 42H, CH_3), 1.78 – 1.90 (m, 9H, CH_2 and C*H*), 2.87 (t, 2H, J_{HH} = 7.3 Hz, SCH_2), 6.14 (dd, 1H, J_{HH} = 15.1, 7.6 Hz, C*H*), 7.56 (d, 1H, J_{HH} = 15.1 Hz, C*H*), 9.84 (d, 1H, J_{HH} = 7.6 Hz, C*H*); ^{13}C NMR (100 MHz, $CDCl_3$, 296K): δ (ppm) 11.62 (CH_2), 22.29 (CH_2), 22.47 (CH_2), 22.50 (CH_2), 23.84 (*C*H), 23.90 (*C*H), 25.67 (CH_3), 35.06 (SCH_2), 126.04, 156.31, 189.56 (*C*O); ^{29}Si NMR (100 MHz, $CDCl_3$, 296K): δ (ppm) -67.56, -67.84, -68.66; MS (ESI+): m/z 967 $[M+Na]^+$; HRMS (ESI+): calcd. for $C_{34}H_{72}NaO_{13}SSi_8$: m/z 967.2745; found: 967.2742; FT-IR (ATR, cm^{-1}): 3050 (=C-H), 3000 ν(C-H), 2820 and 2880 ν(O=**C-H**), 1690 ν(C=O), 1500 (C=C), 1450, 1380 asymmetric and symmetric δ(CH_2), 1100 ν(Si -O), 850 δ(Si-O), 750 ν(Si-C) and ν(S-C), 500 δ(Si-O) and δ(S-C).

2. FT-IR spectra of products P1, P9 and P12

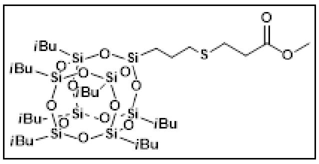

Produkt P1

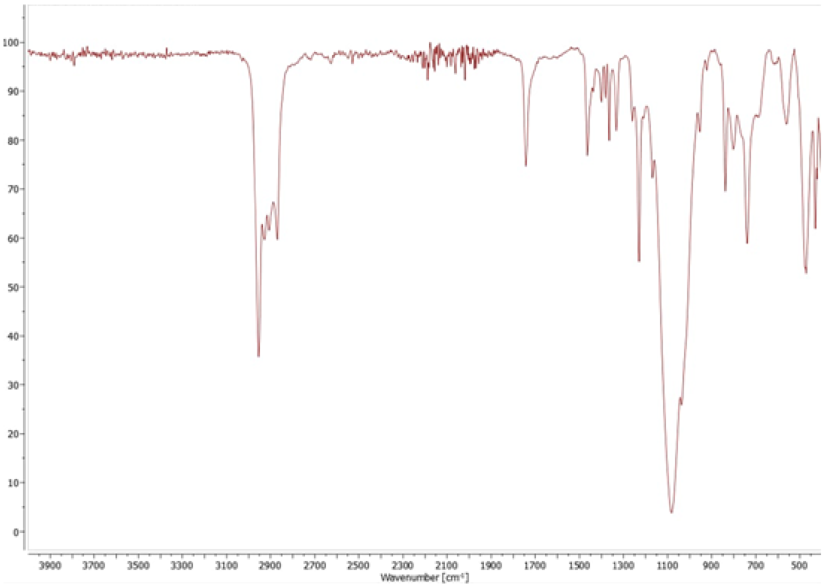

Figure S1. FT-IR spectra of product **P1**

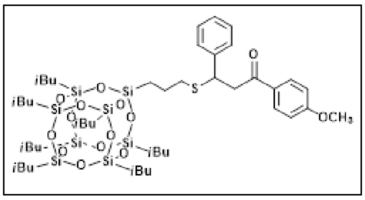

Produkt P9

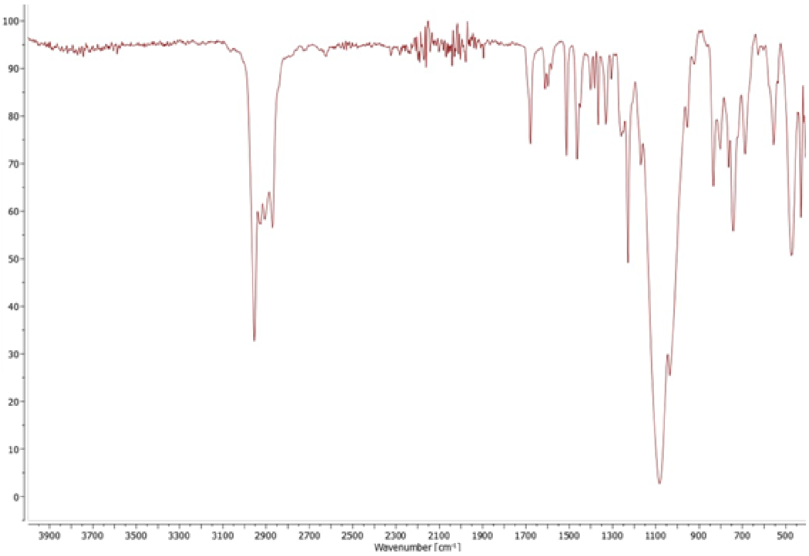

Figure S2. FT-IR spectra of product **P9**

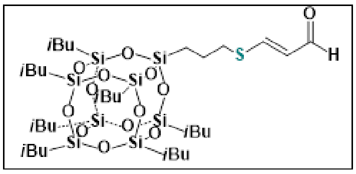

Produkt P12

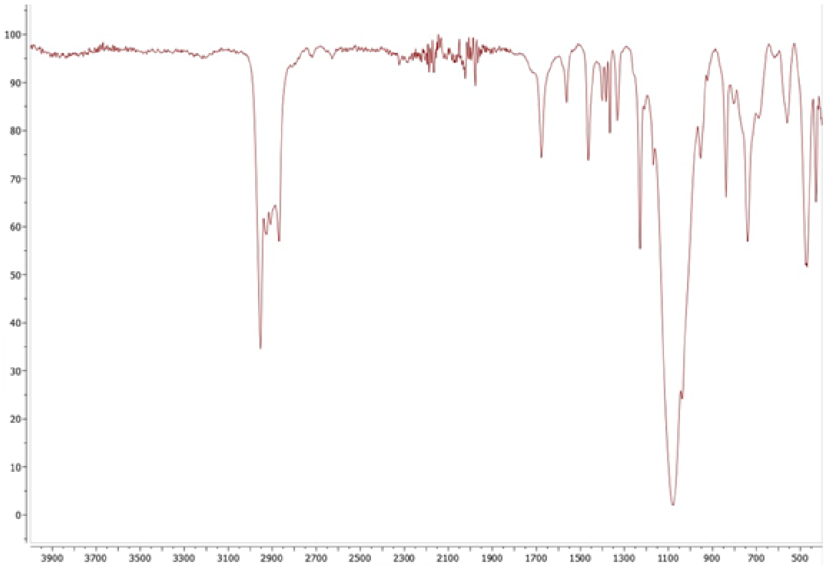

Figure S3. FT-IR spectra of product **P12**

3. NMR spectra of products P1-P12

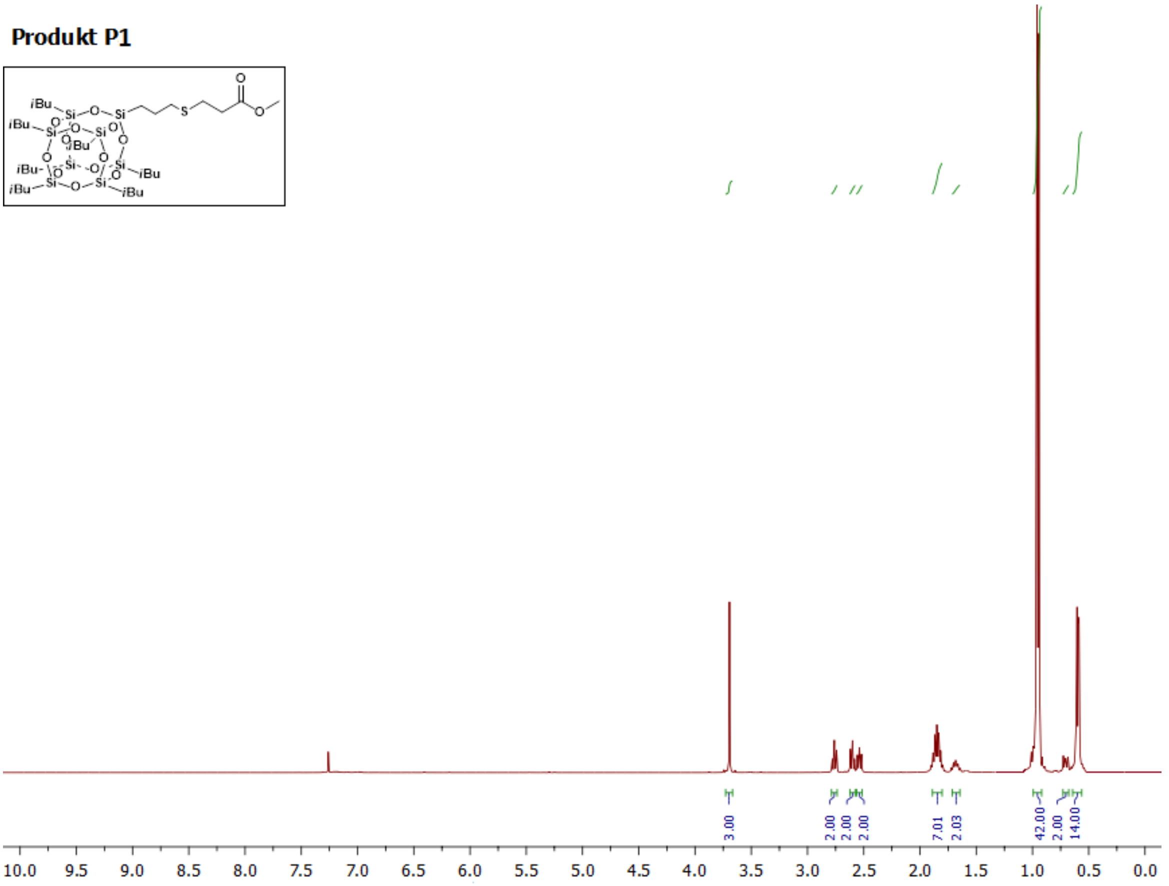

Figure S4. ^{1}H NMR (400 MHz, $CDCl_3$) of product **P1**

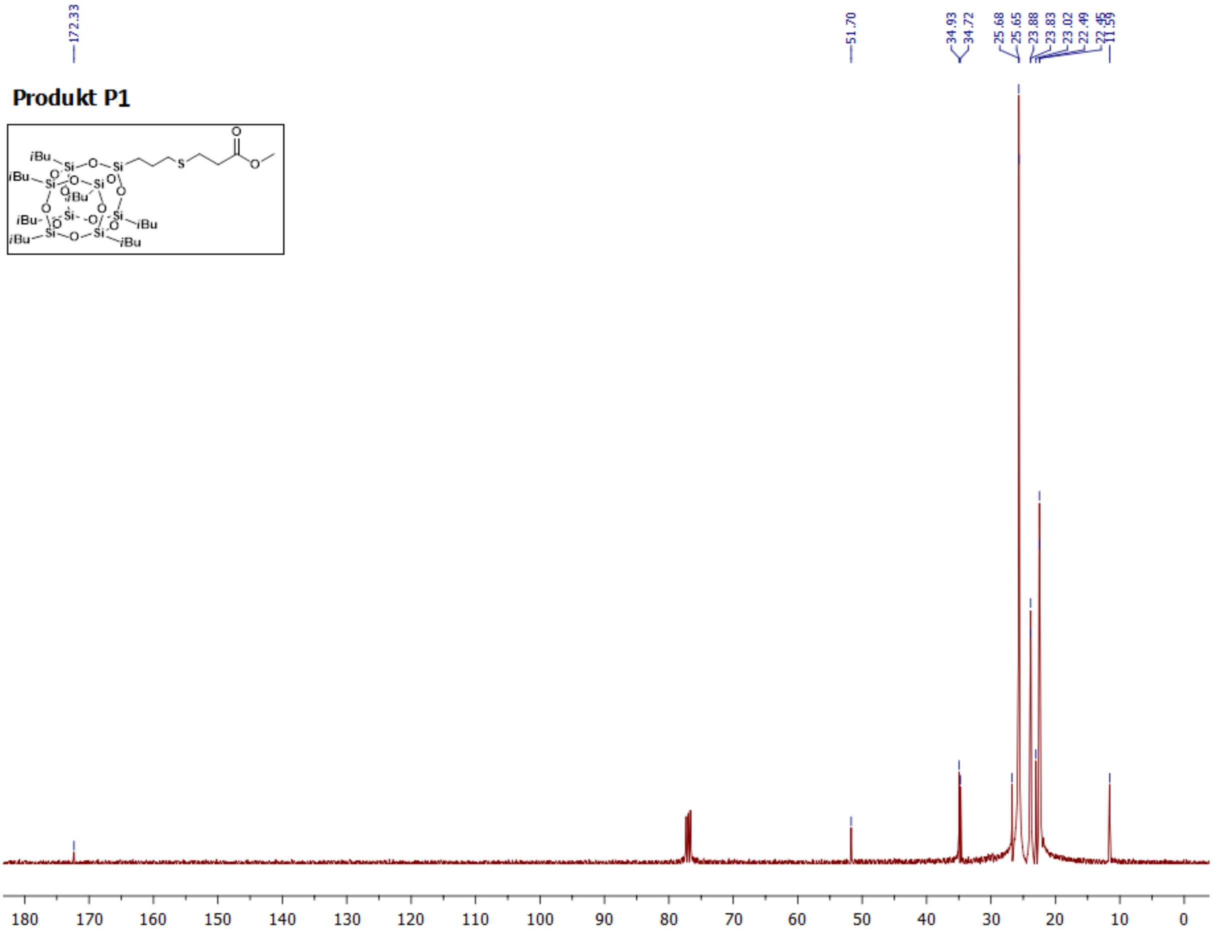

Figure S5. ^{13}C NMR (101 MHz, $CDCl_3$) of product **P1**

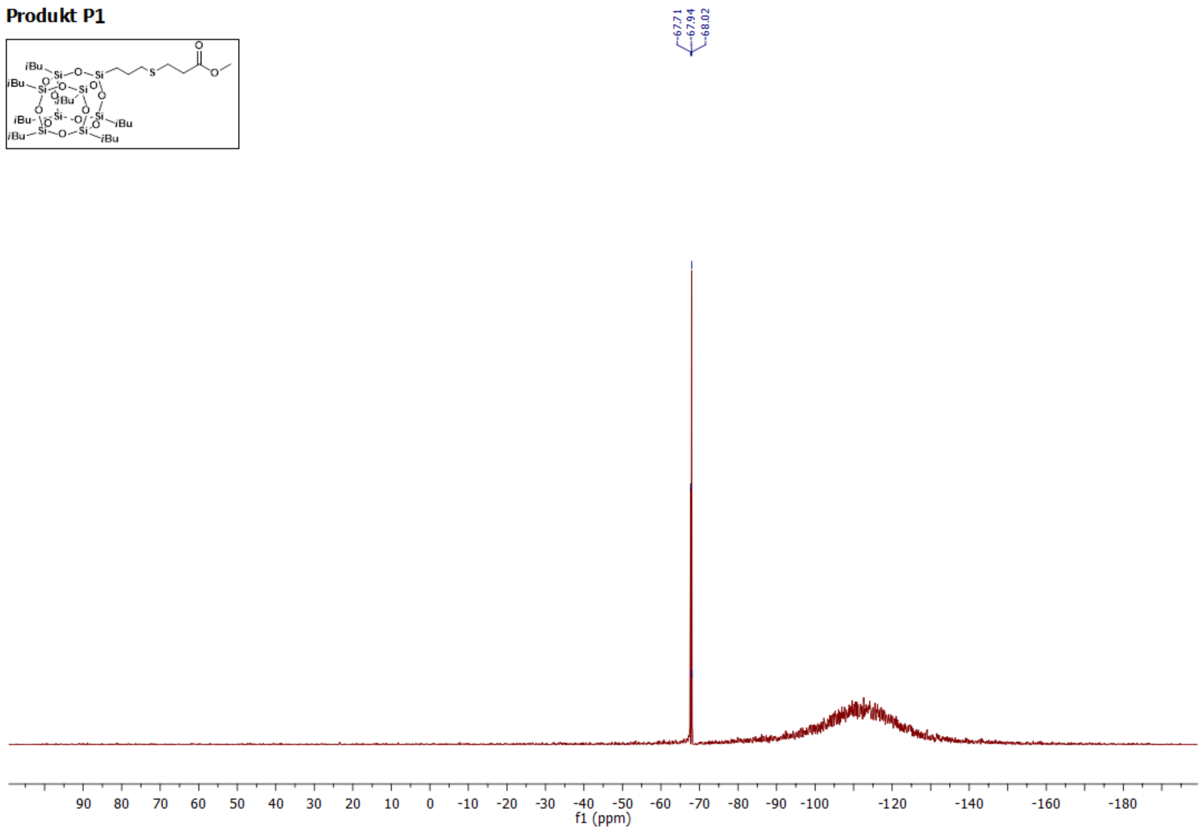

Figure S6. ^{29}Si NMR (79 MHz, $CDCl_3$) of product **P1**

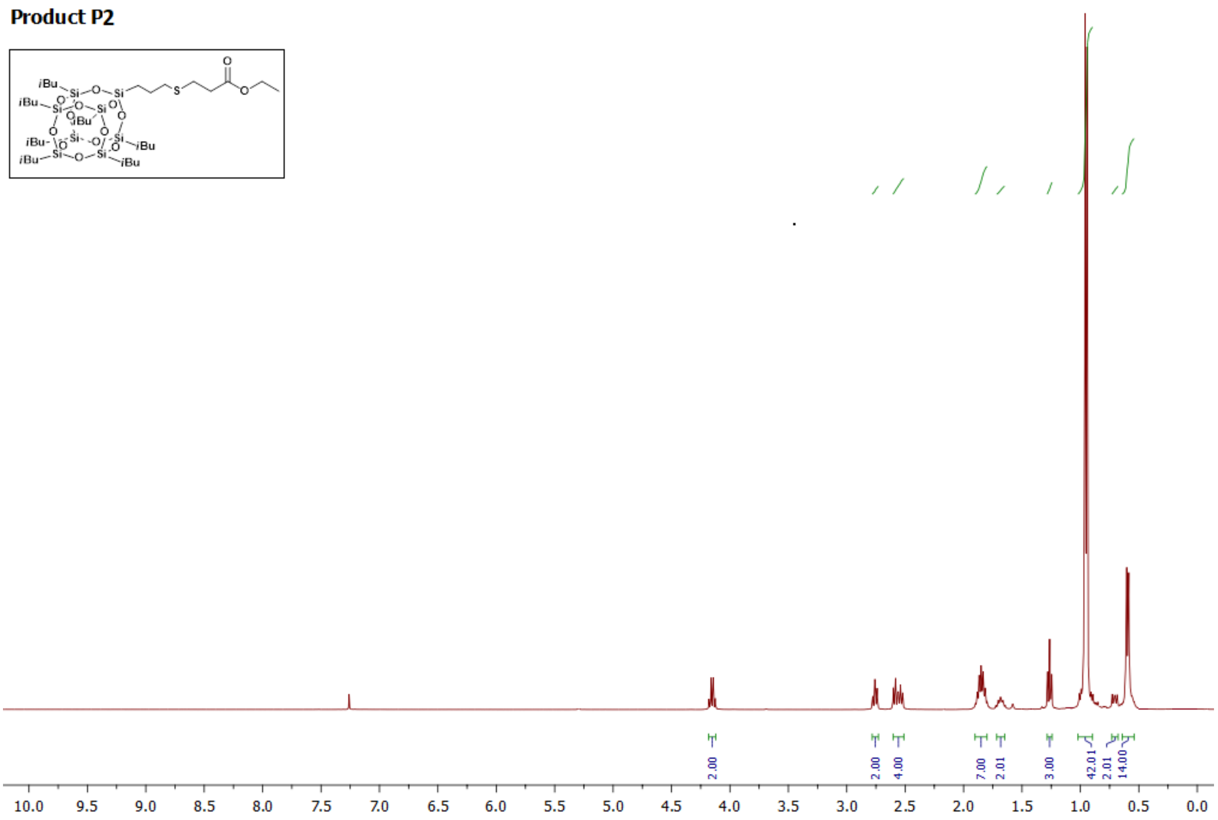

Figure S7. ^{1}H NMR (400 MHz, $CDCl_3$) of product **P2**

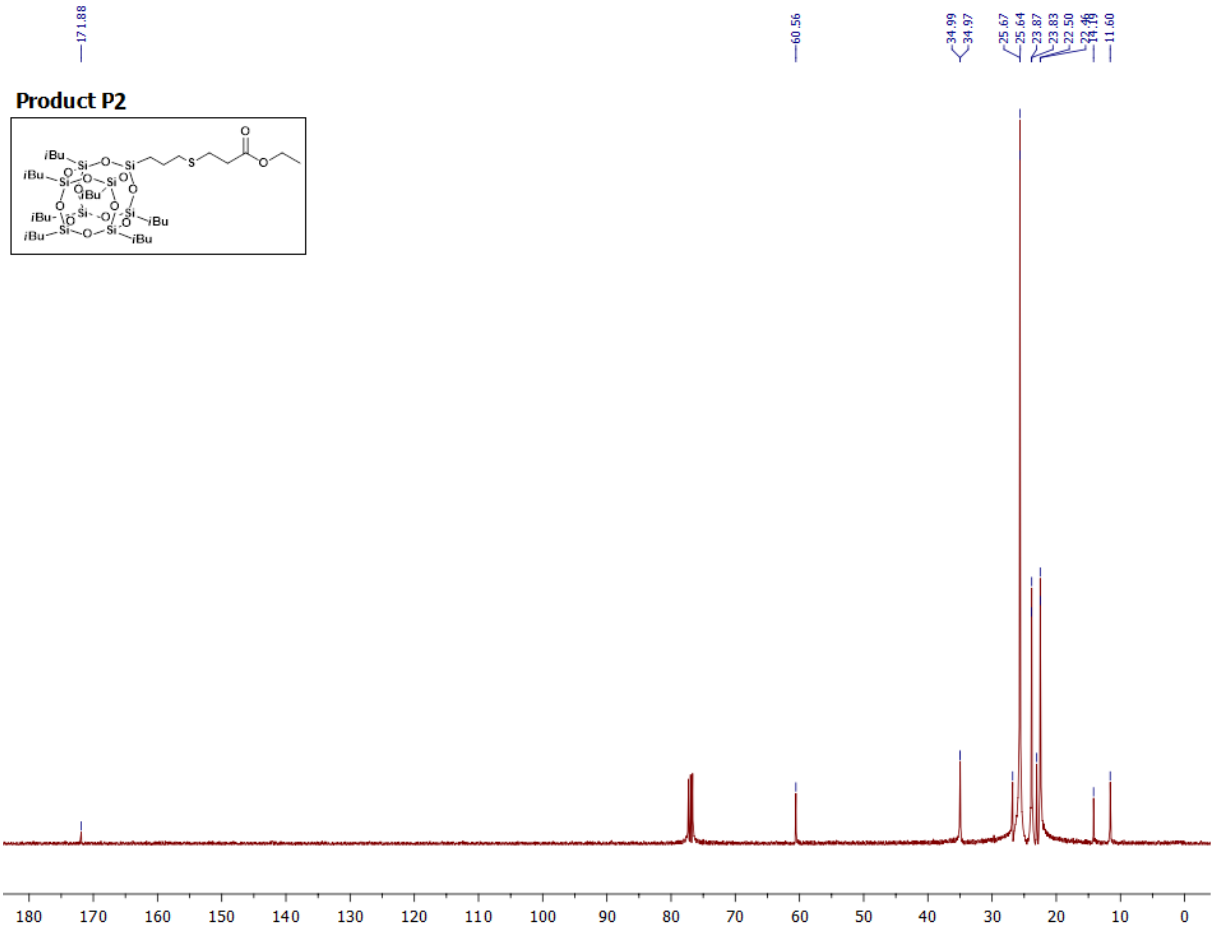

Figure S8. ^{13}C NMR (101 MHz, $CDCl_3$) of product **P2**

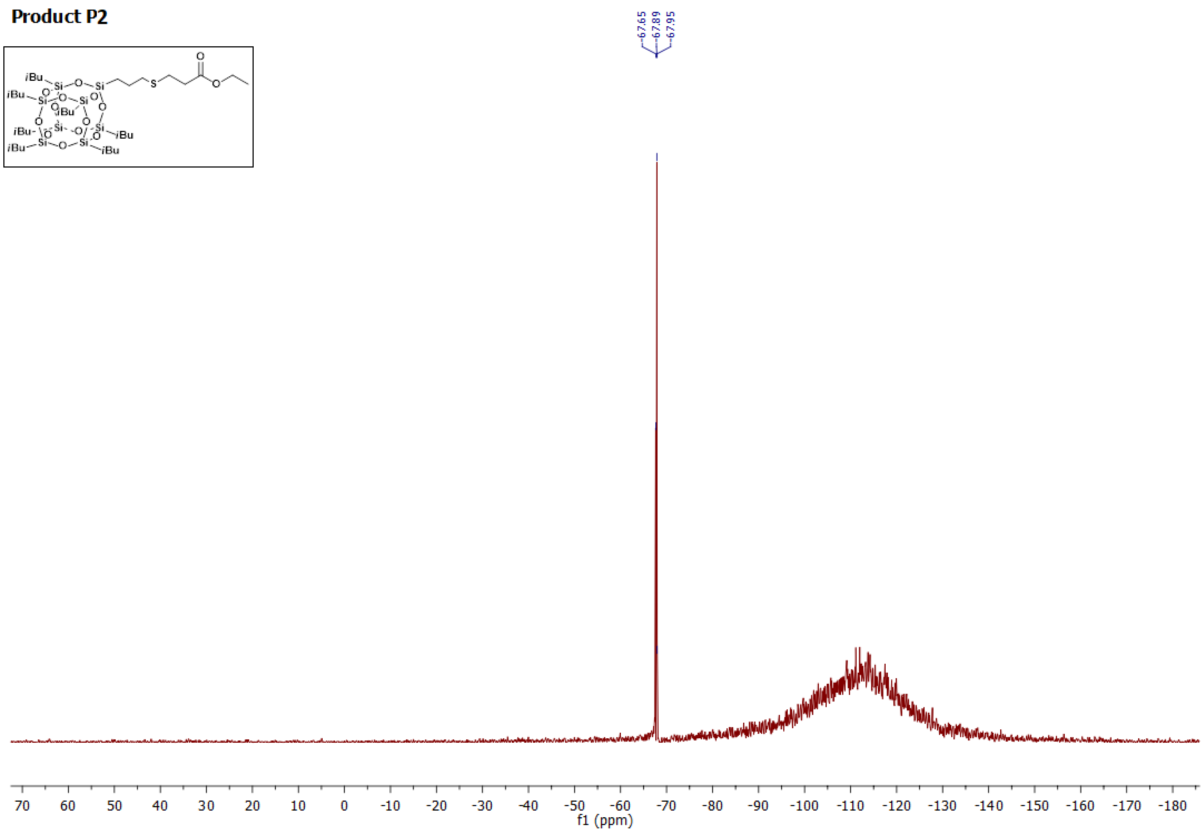

Figure S9. ^{29}Si NMR (79 MHz, $CDCl_3$) of product **P2**

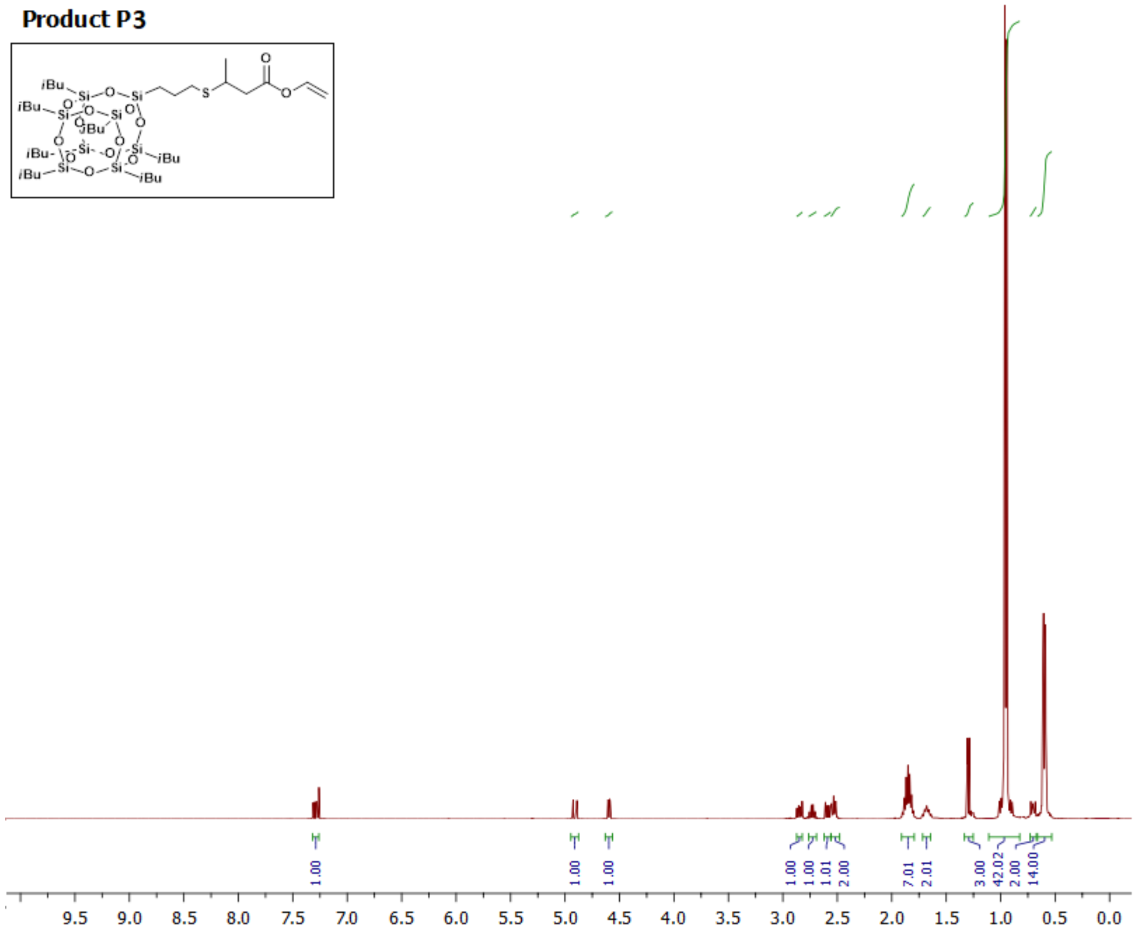

Figure S10. ^{1}H NMR (400 MHz, $CDCl_3$) of product **P3**

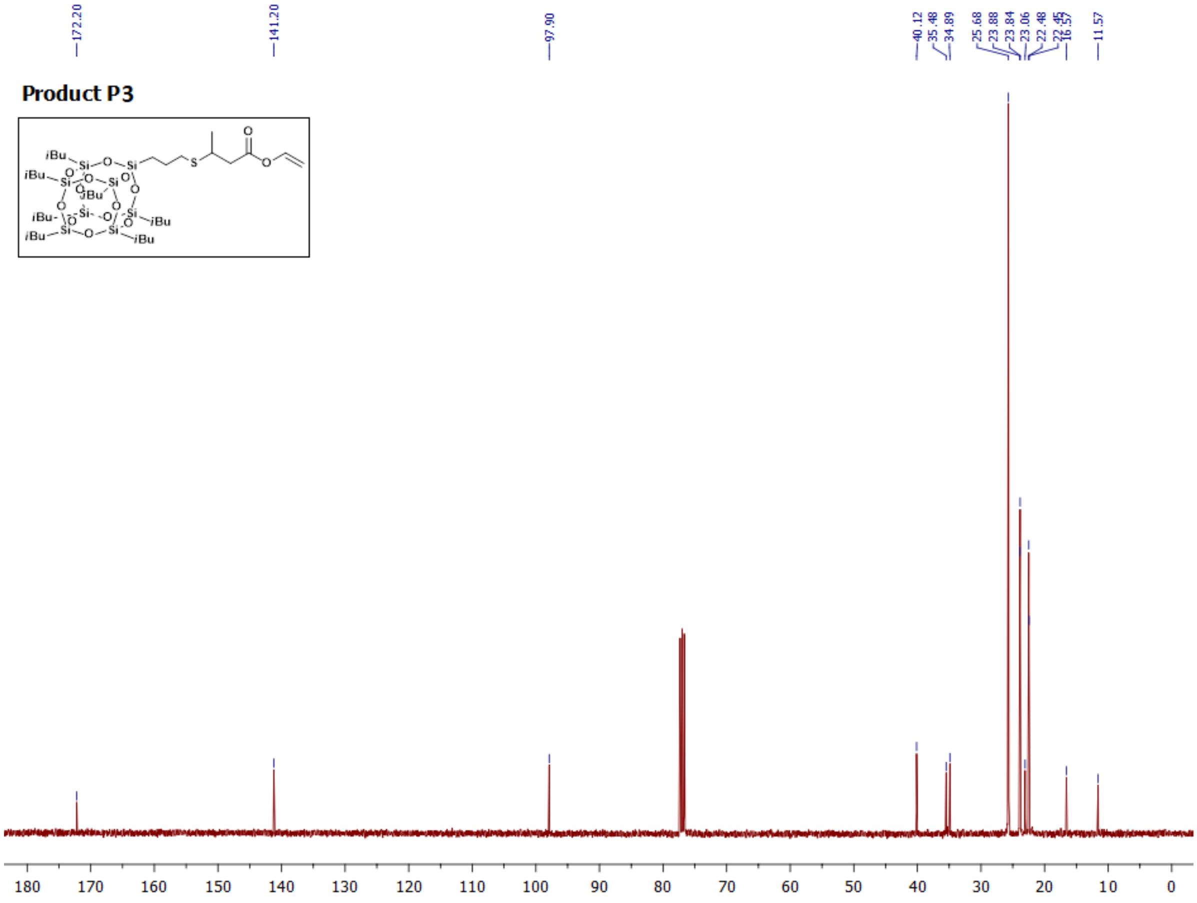

Figure S11. ^{13}C NMR (101 MHz, $CDCl_3$) of product **P3**

Product P3

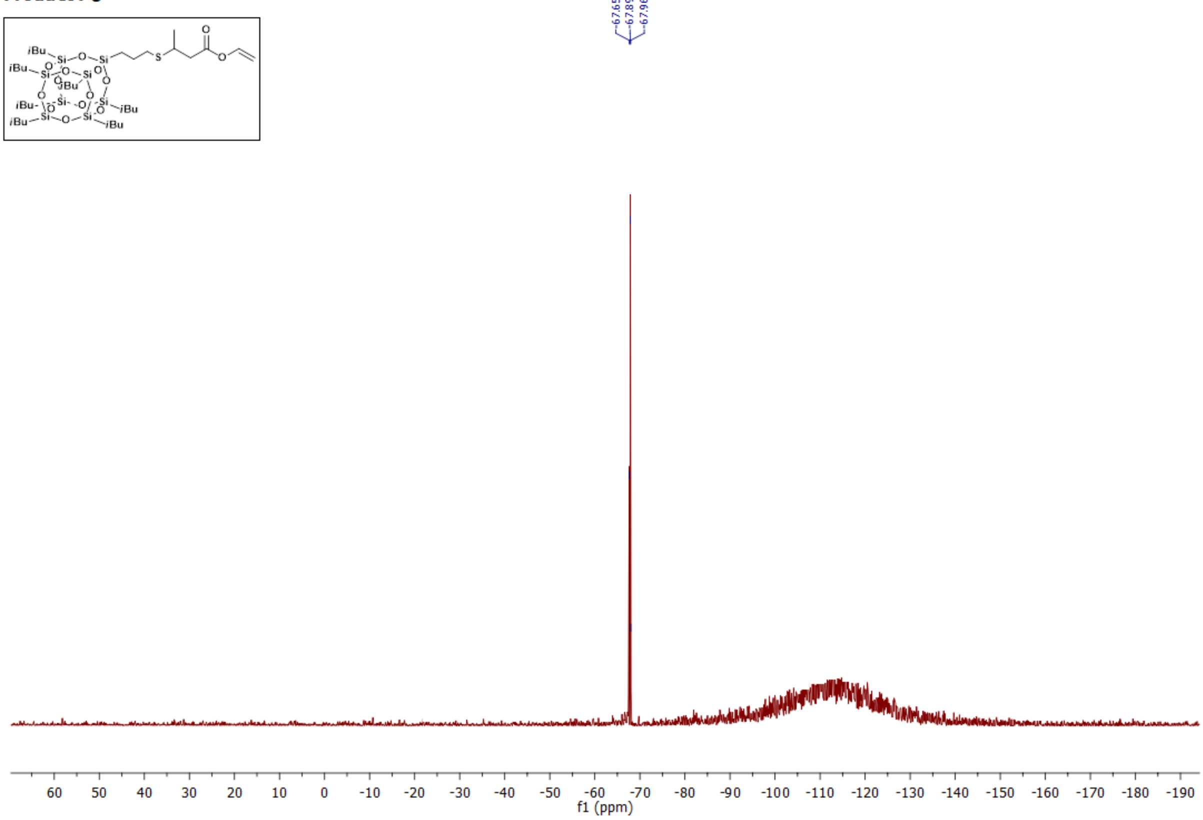

Figure S12. ^{29}Si NMR (79 MHz, $CDCl_3$) of product **P3**

Product P4

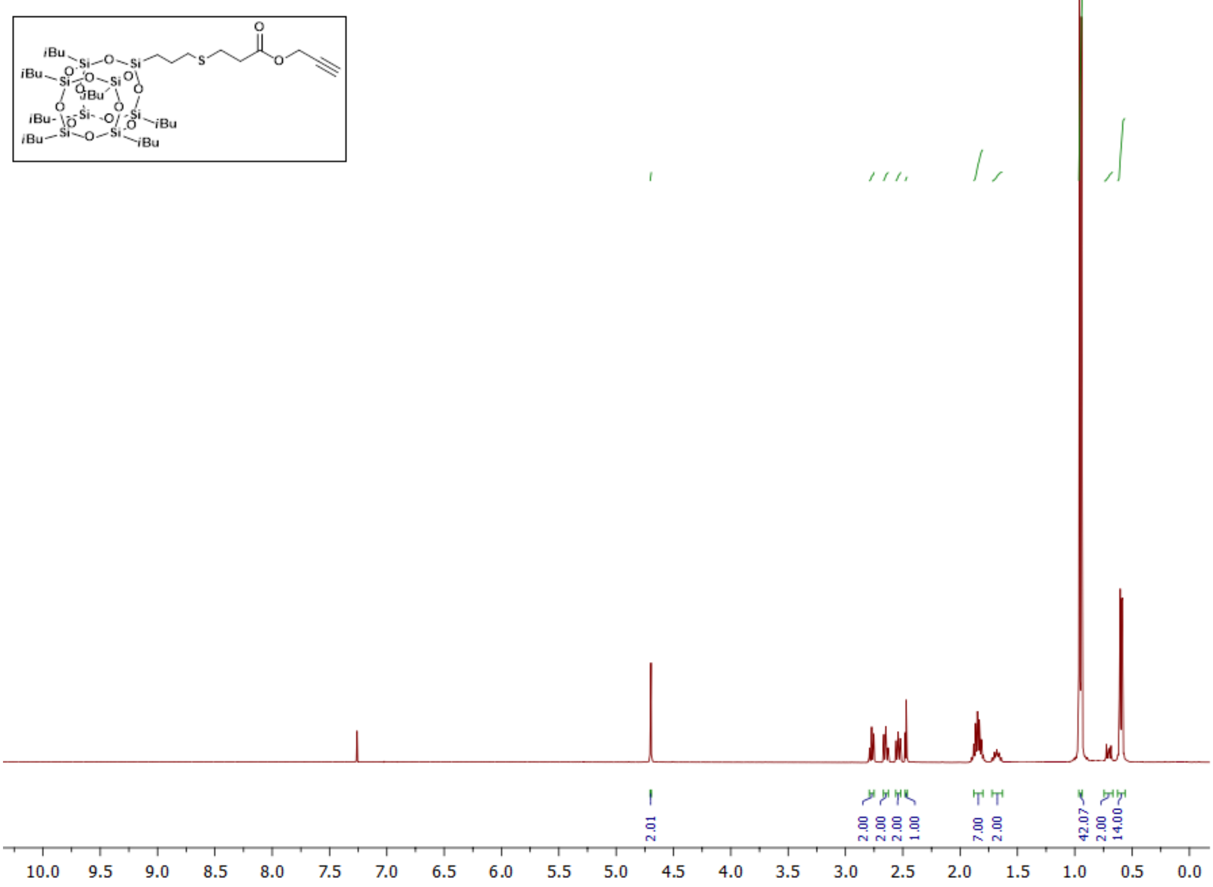

Figure S13. ^{1}H NMR (400 MHz, $CDCl_3$) of product **P4**

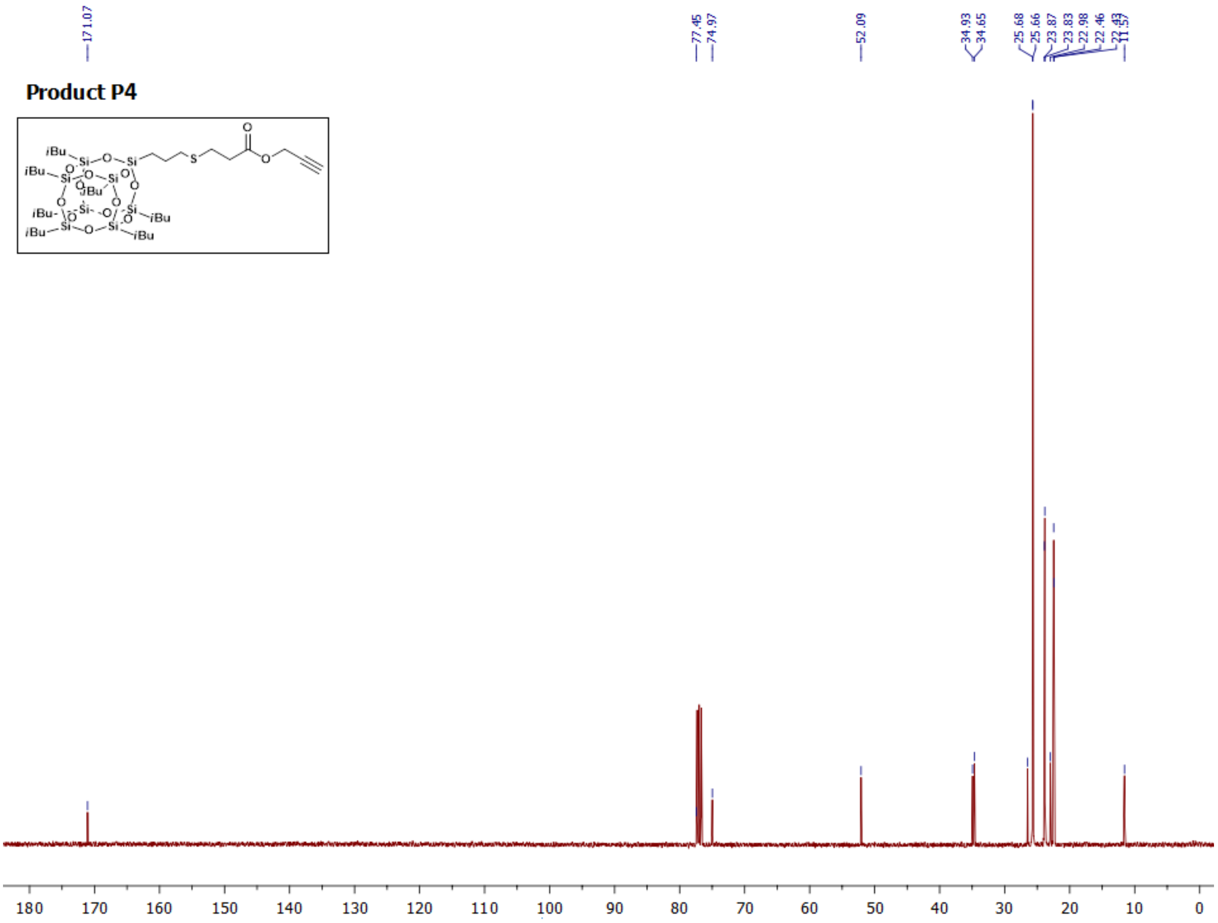

Figure S14. ^{13}C NMR (101 MHz, $CDCl_3$) of product **P4**

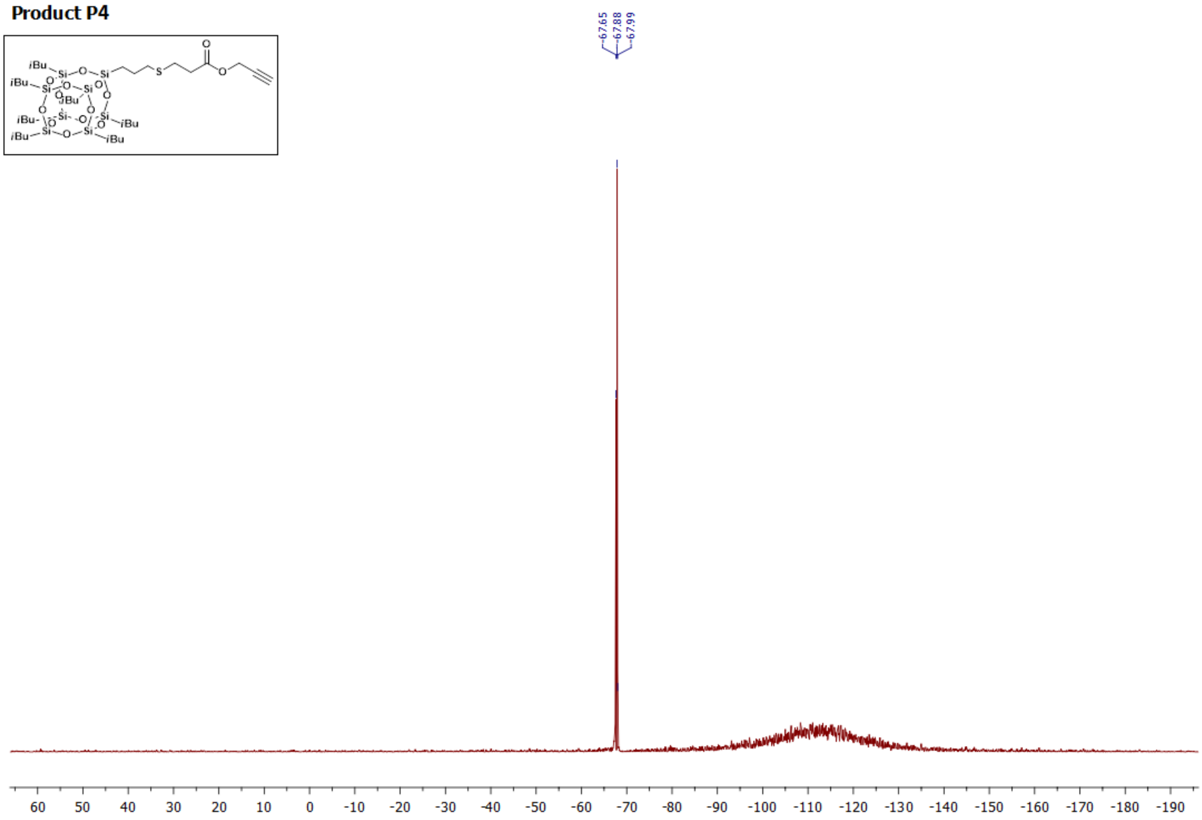

Figure S15. ^{29}Si NMR (79 MHz, $CDCl_3$) of product **P4**

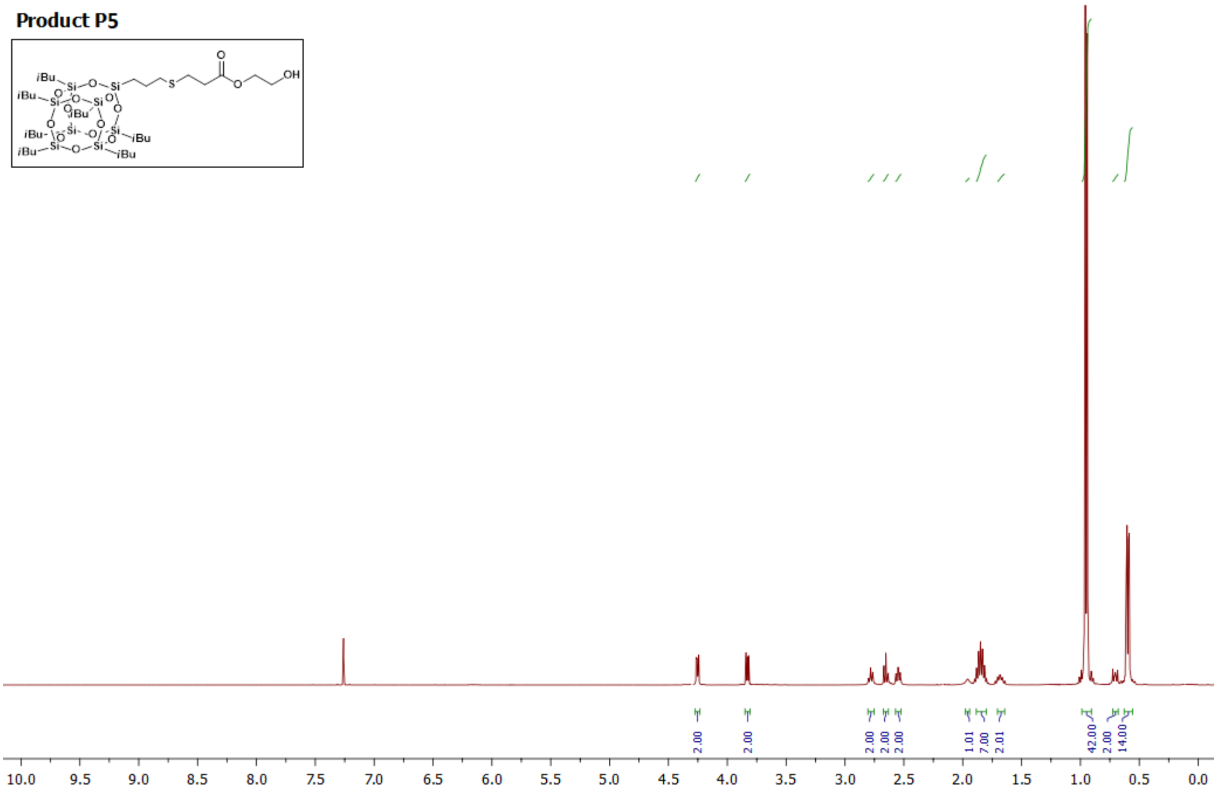

Figure S16. ${}^{1}H$ NMR (400 MHz, $CDCl_3$) of product **P5**

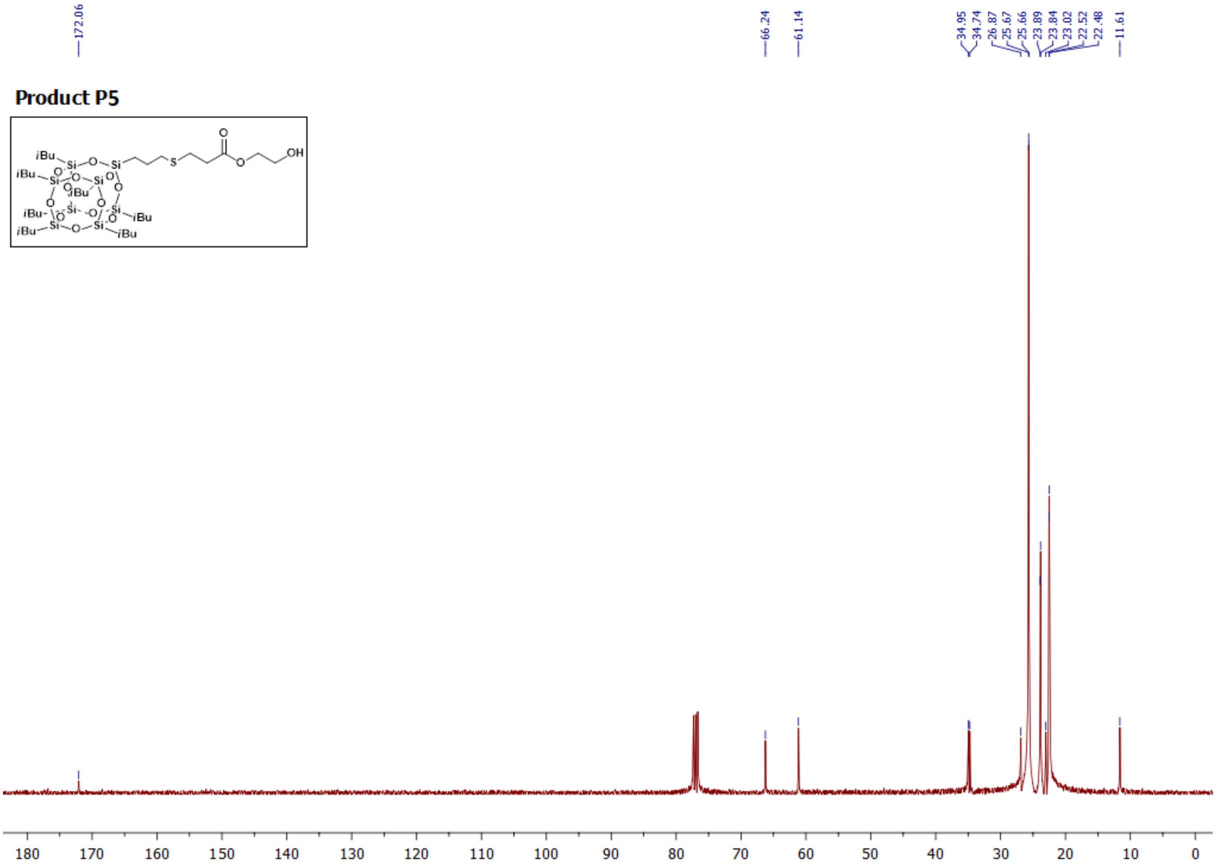

Figure S17. ${}^{13}C$ NMR (101 MHz, $CDCl_3$) of product **P5**

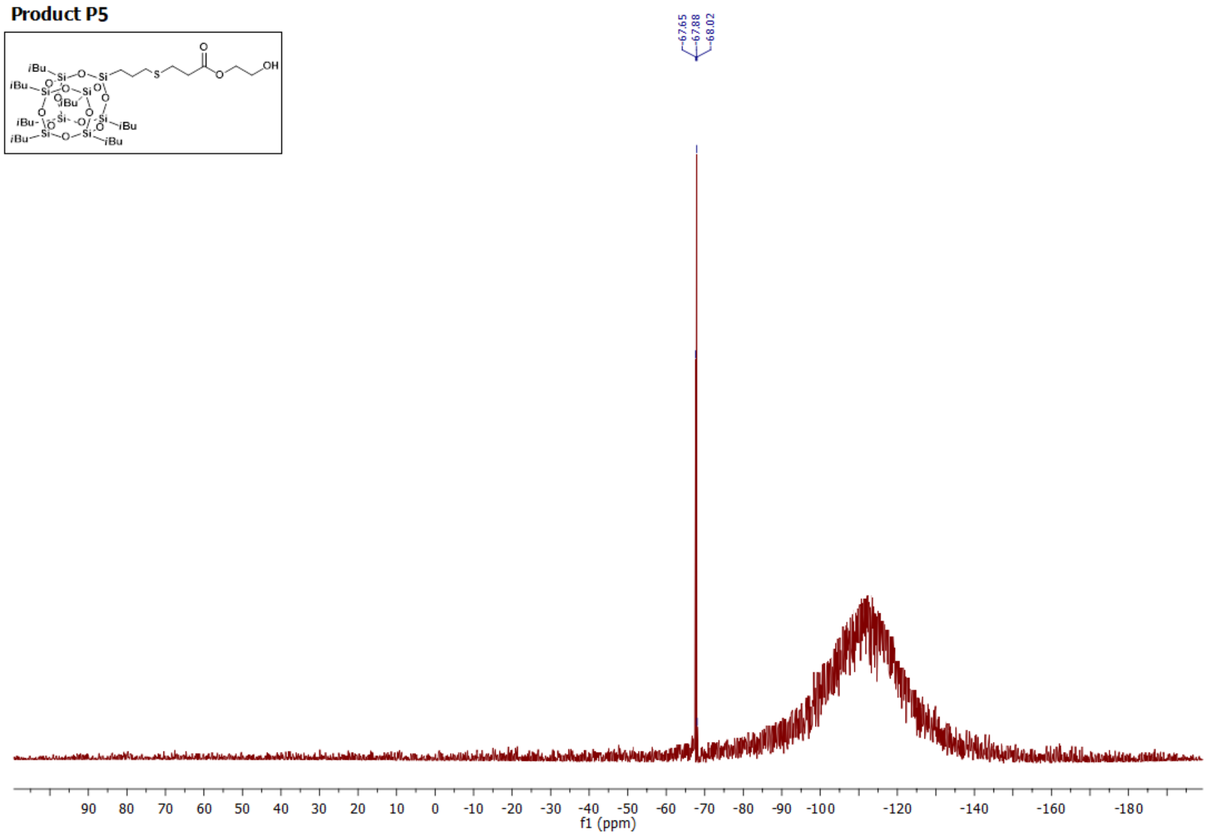

Figure S18. ^{29}Si NMR (79 MHz, $CDCl_3$) of product **P5**

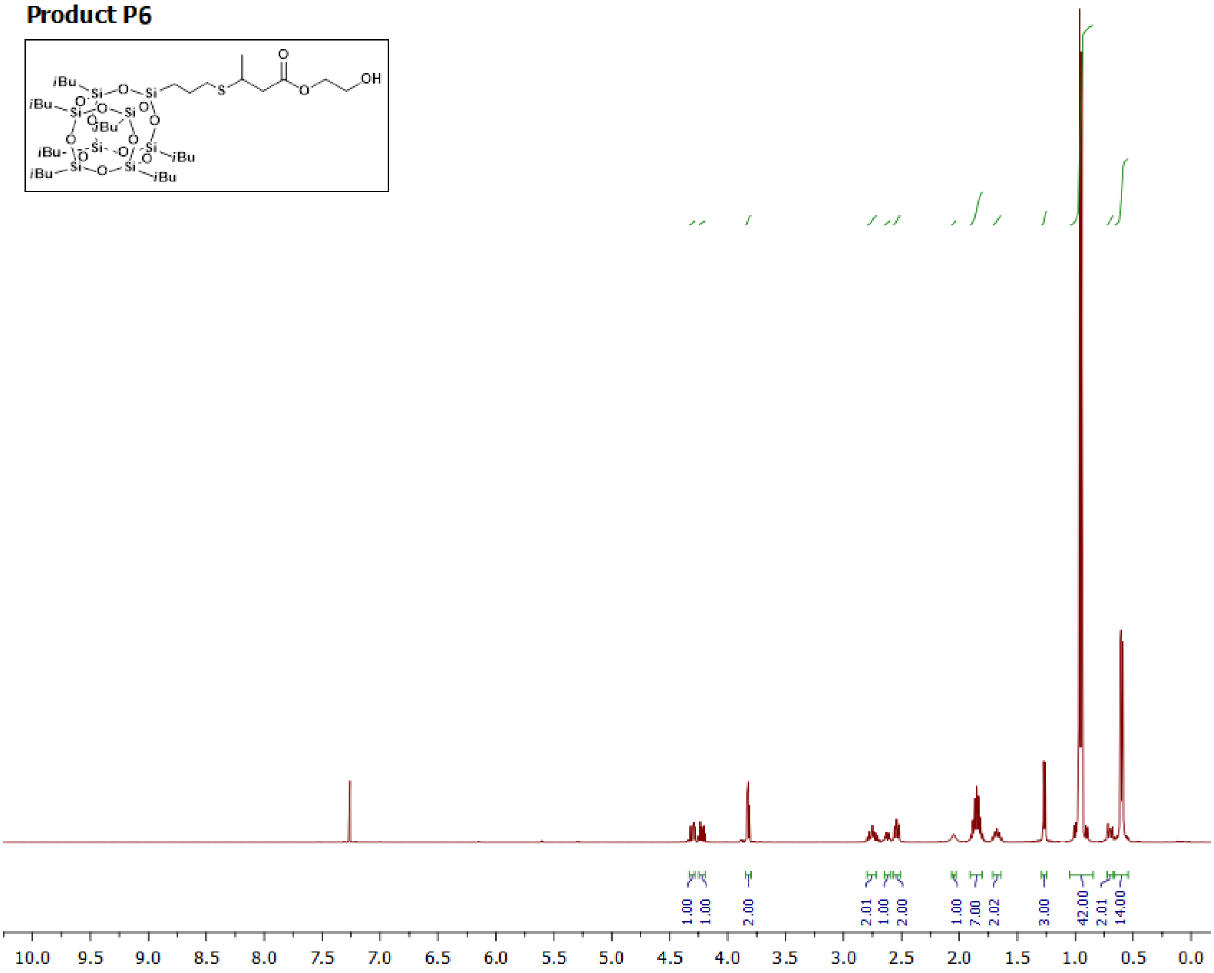

Figure S19. ^{1}H NMR (400 MHz, $CDCl_3$) of product **P6**

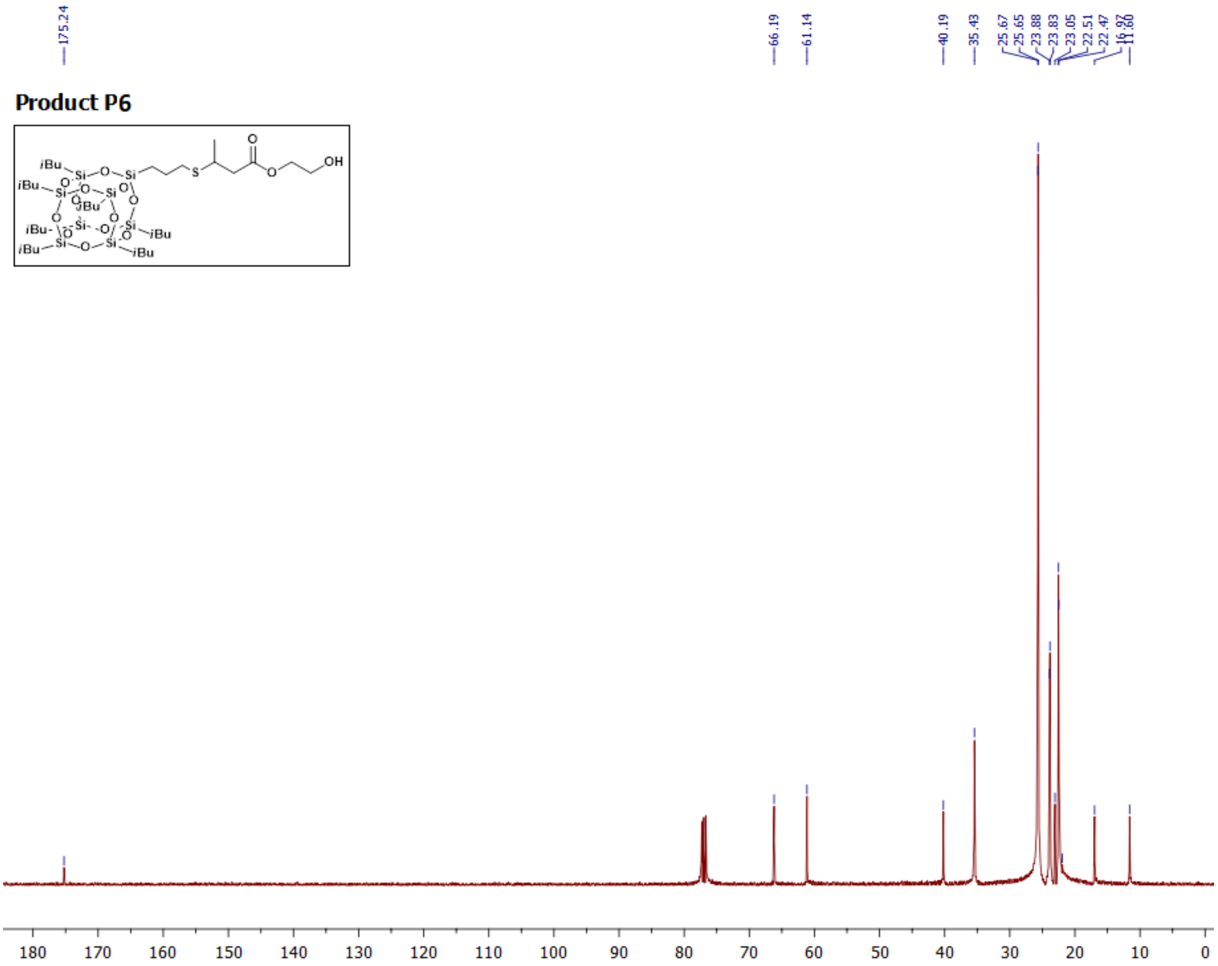

Figure S20. ^{13}C NMR (101 MHz, $CDCl_3$) of product **P6**

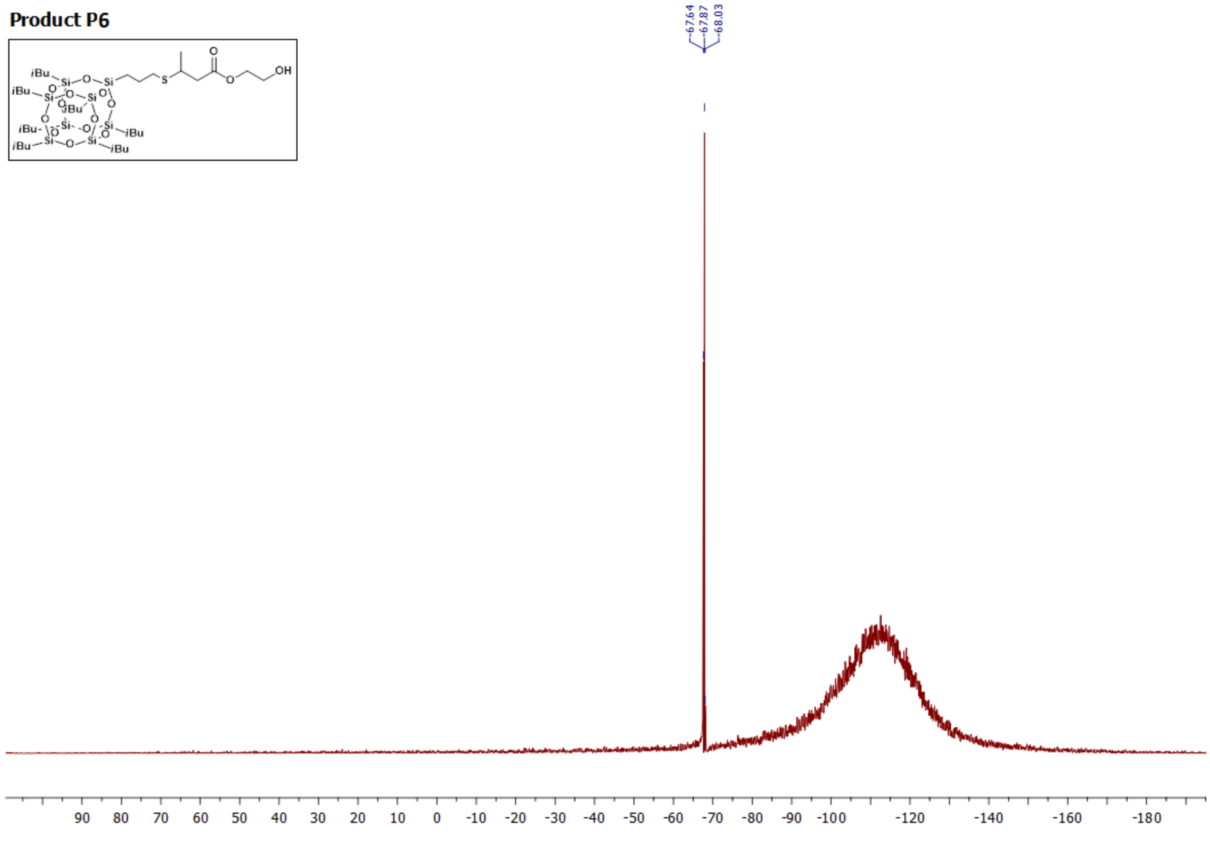

Figure S21. ^{29}Si NMR (79 MHz, $CDCl_3$) of product **P6**

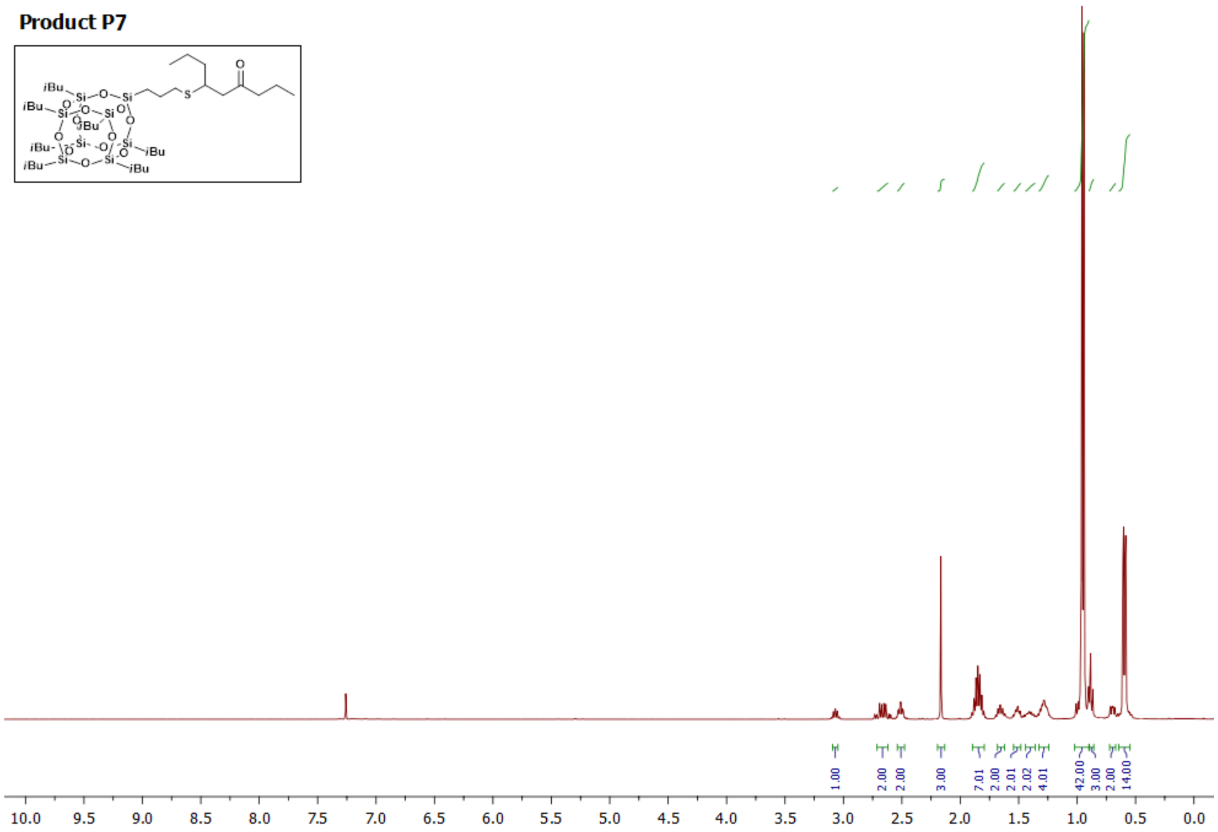

Figure S22. ¹H NMR (400 MHz, $CDCl_3$) of product **P7**

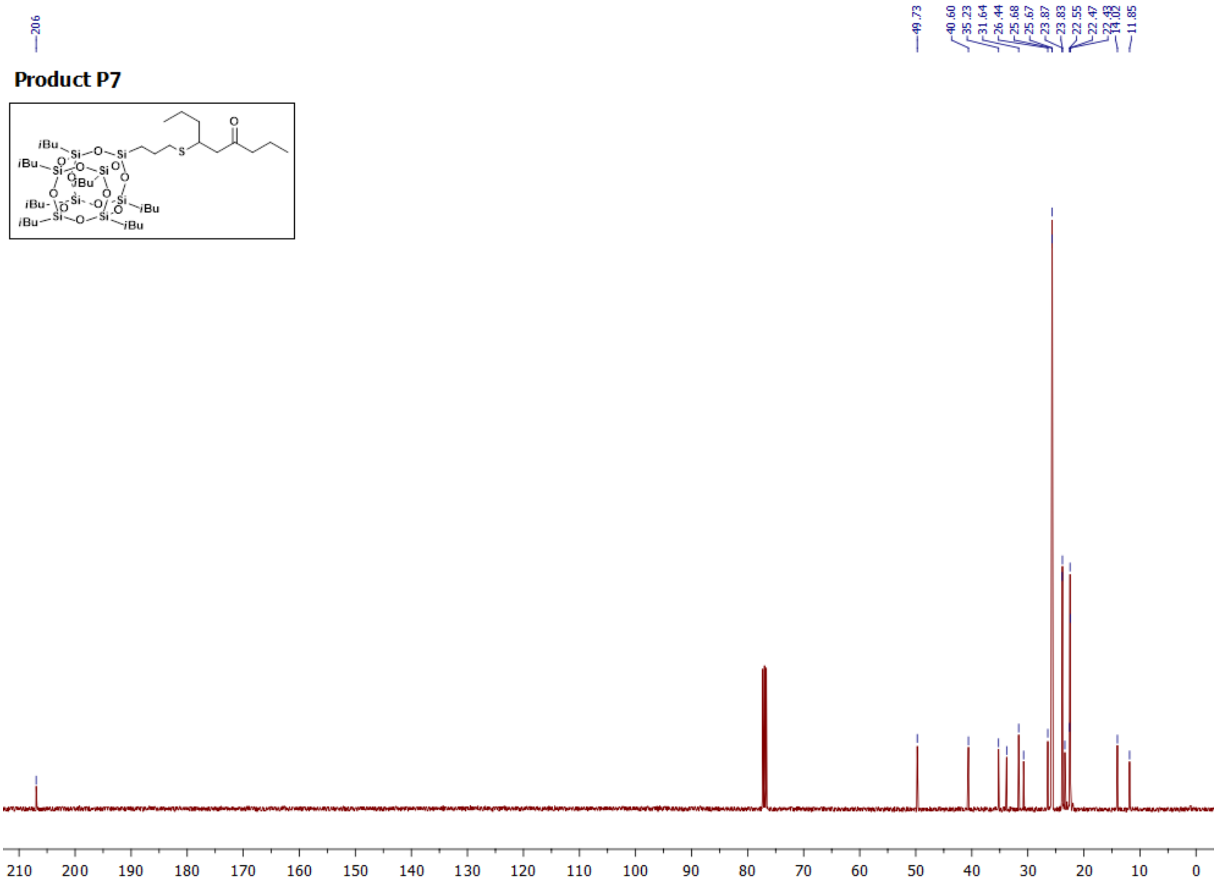

Figure S23. ¹³C NMR (101 MHz, $CDCl_3$) of product **P7**

Product P7

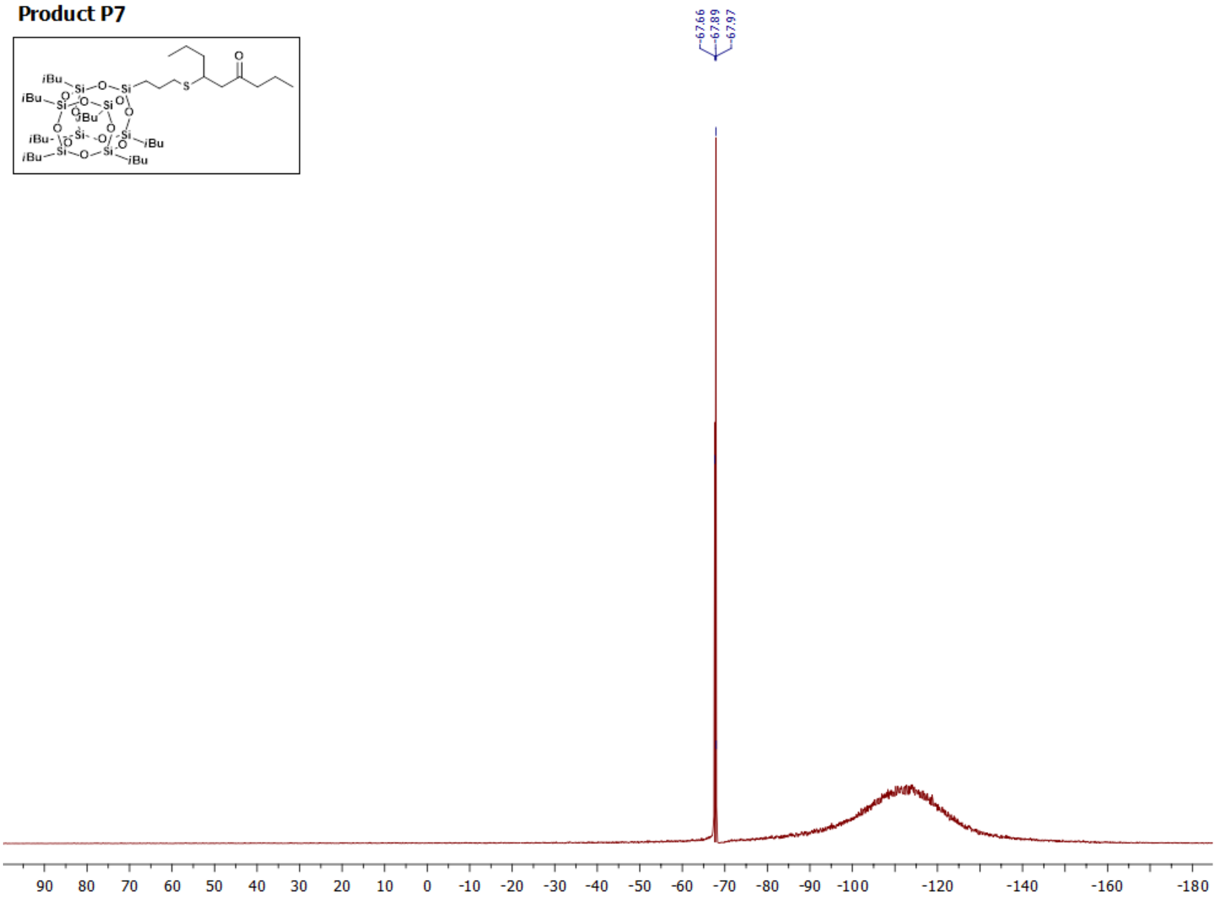

Figure S24. ^{29}Si NMR (79 MHz, $CDCl_3$) of product **P7**

Product P8

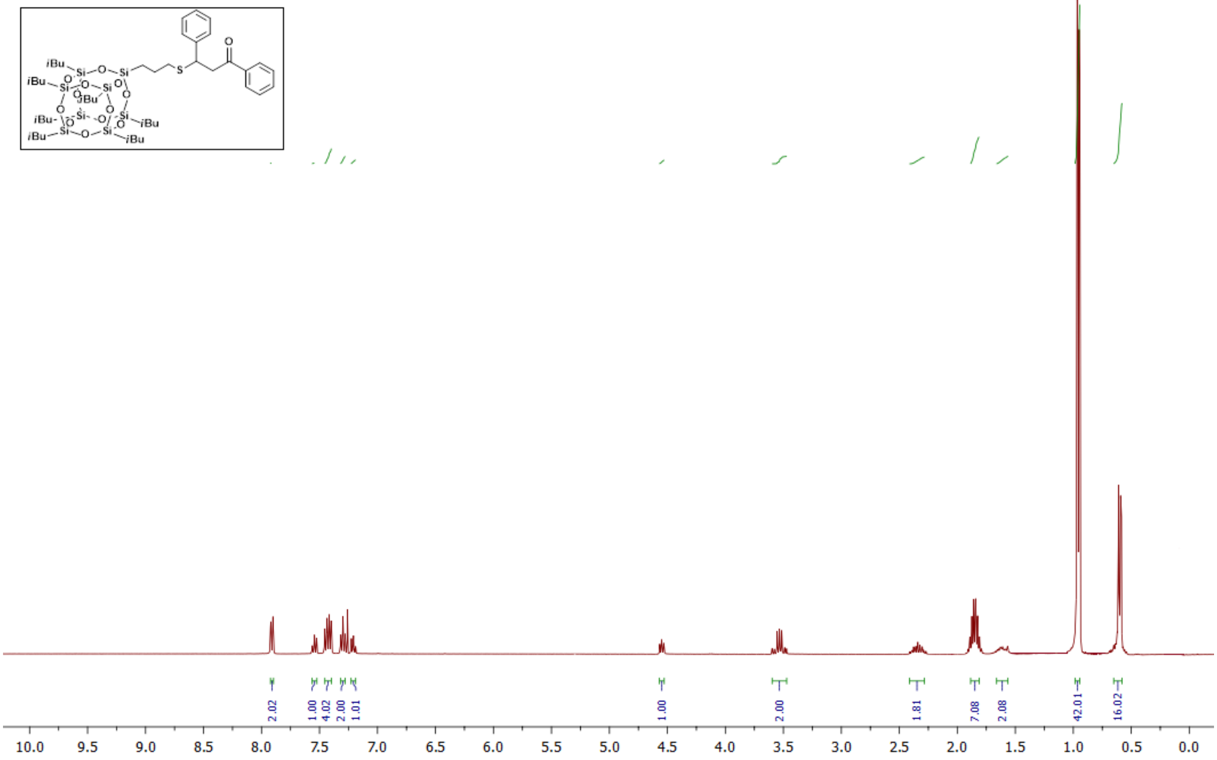

Figure S25. ^{1}H NMR (400 MHz, $CDCl_3$) of product **P8**

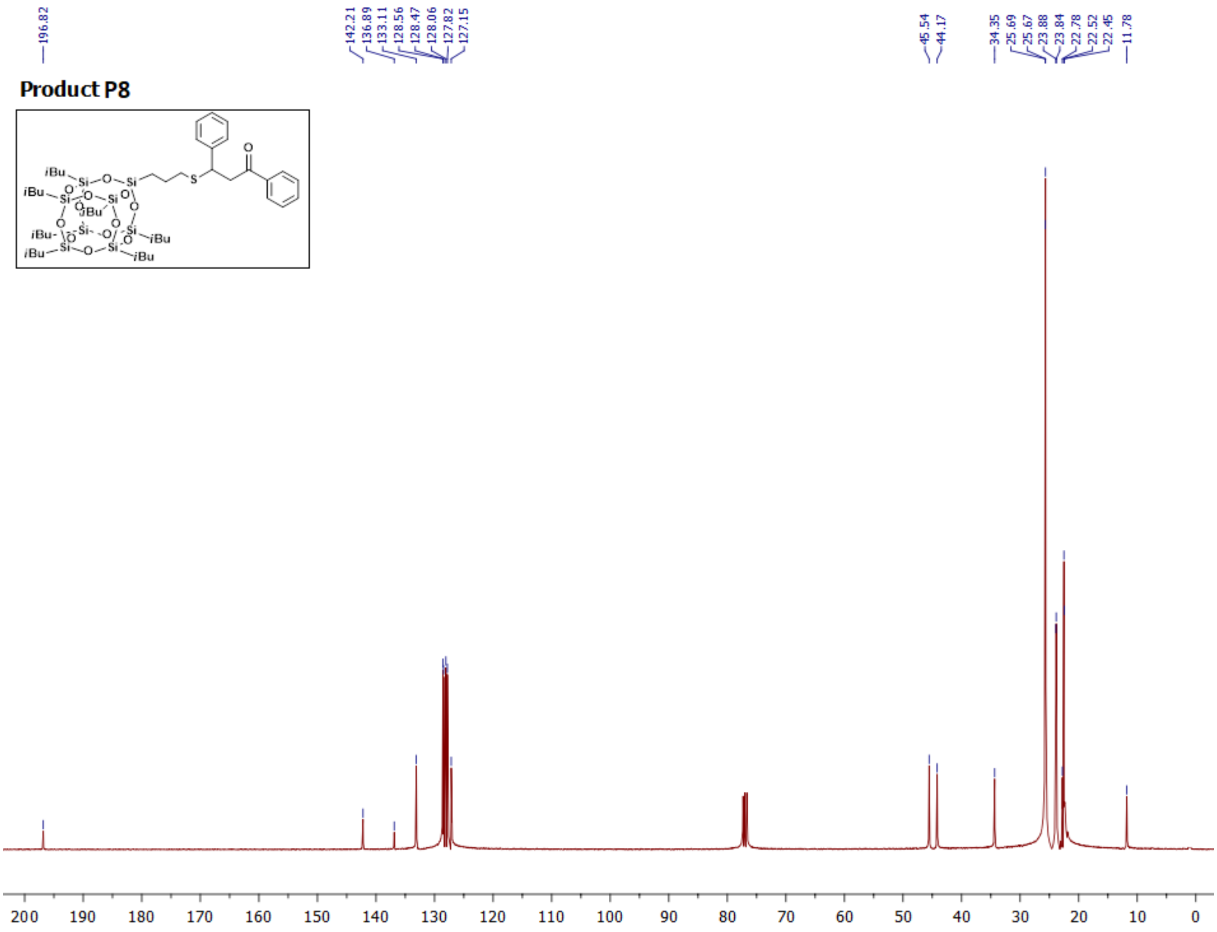

Figure S26. ^{13}C NMR (101 MHz, $CDCl_3$) of product **P8**

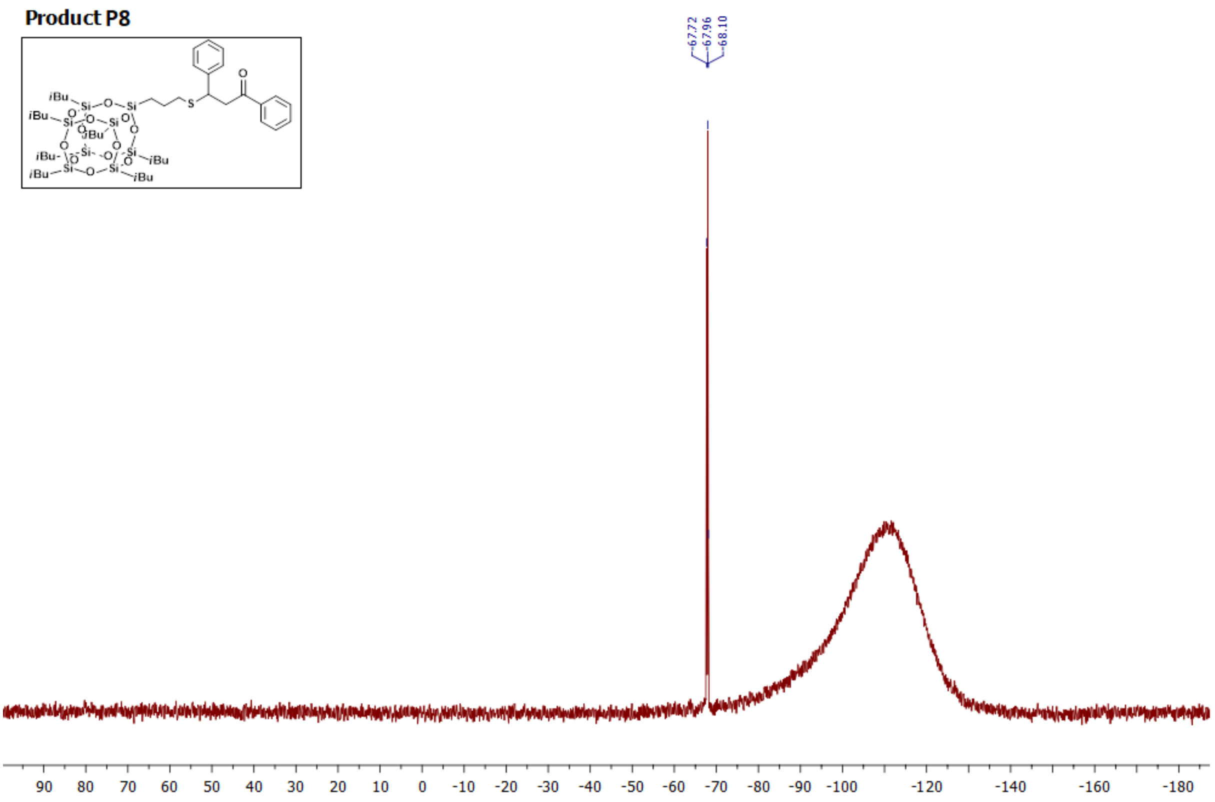

Figure S27. ^{29}Si NMR (79 MHz, $CDCl_3$) of product **P8**

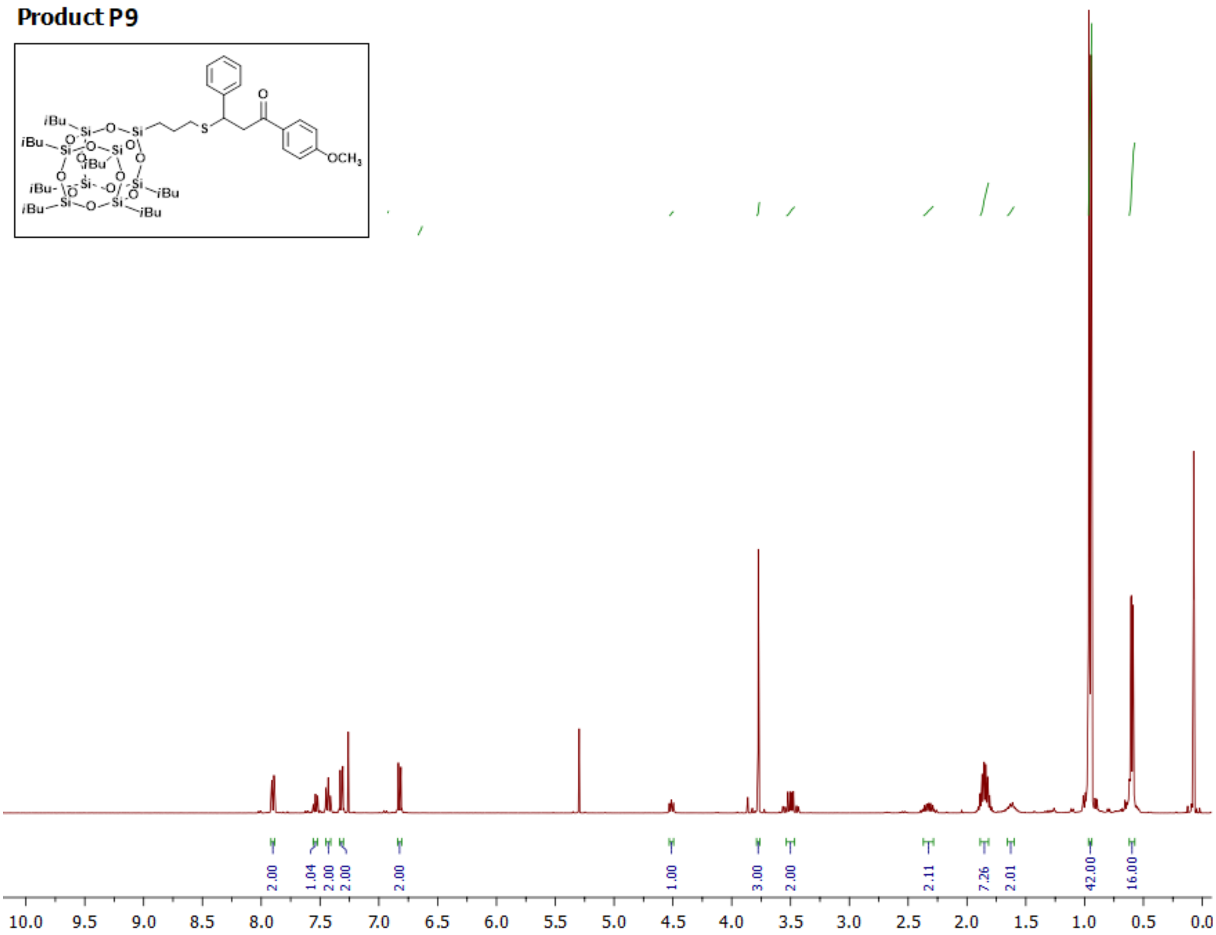

Figure S28. ^{1}H NMR (400 MHz, $CDCl_3$) of product **P9**

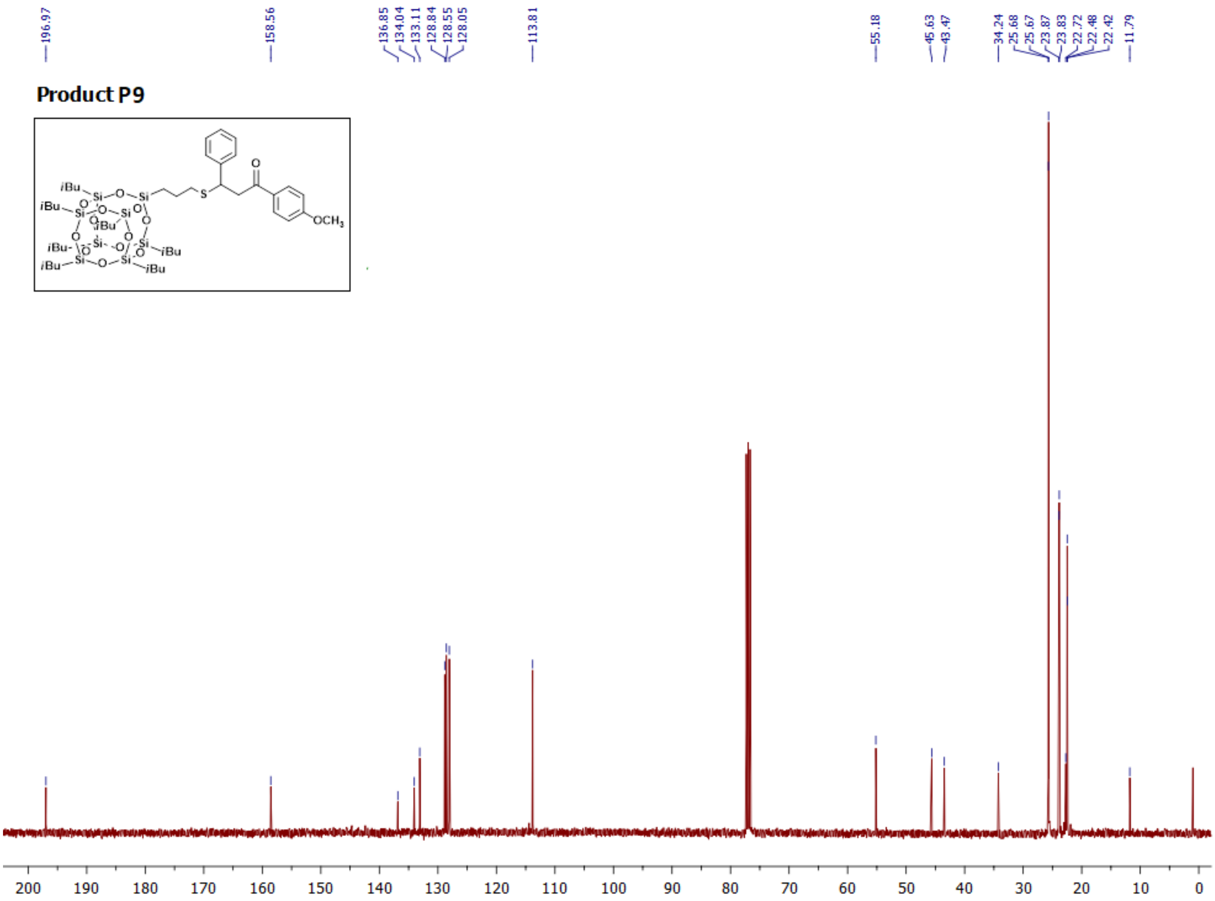

Figure S29. ^{13}C NMR (101 MHz, $CDCl_3$) of product **P9**

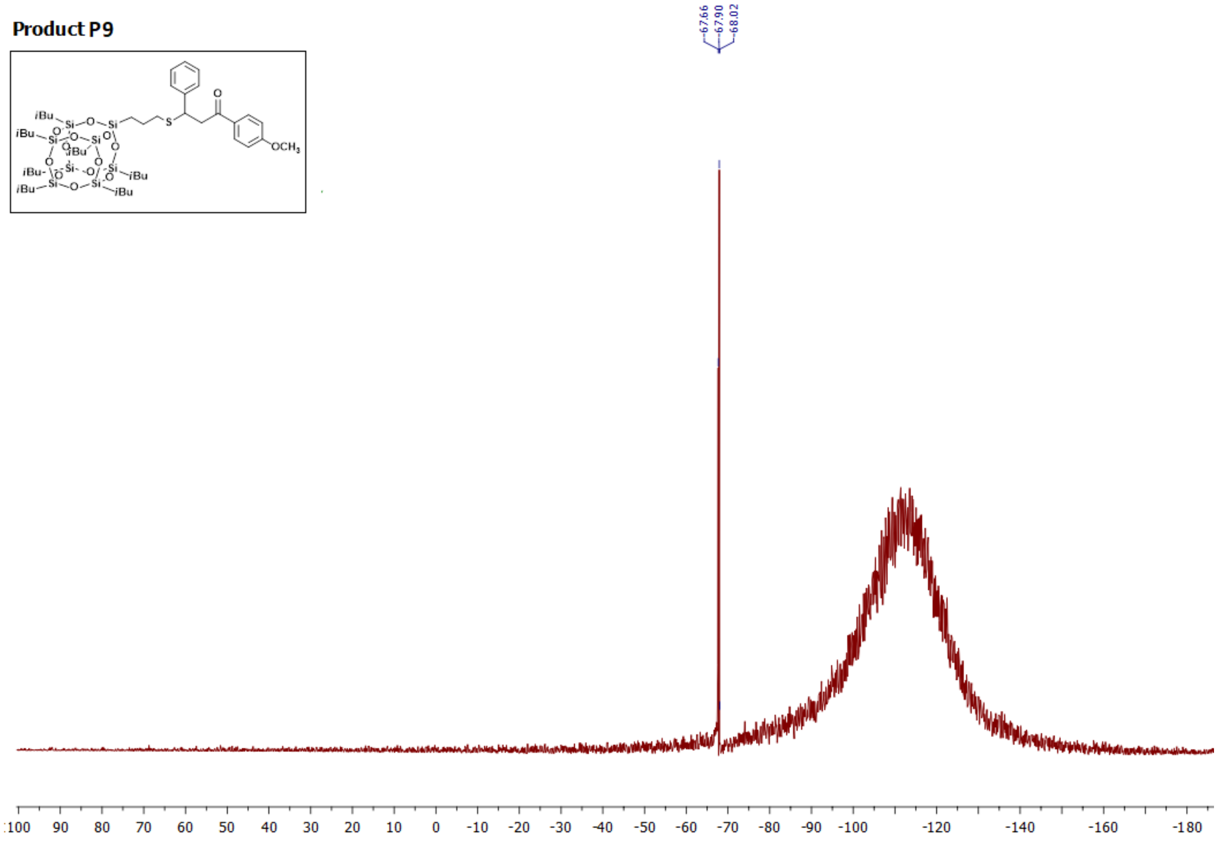

Figure S30. ^{29}Si NMR (79 MHz, $CDCl_3$) of product **P9**

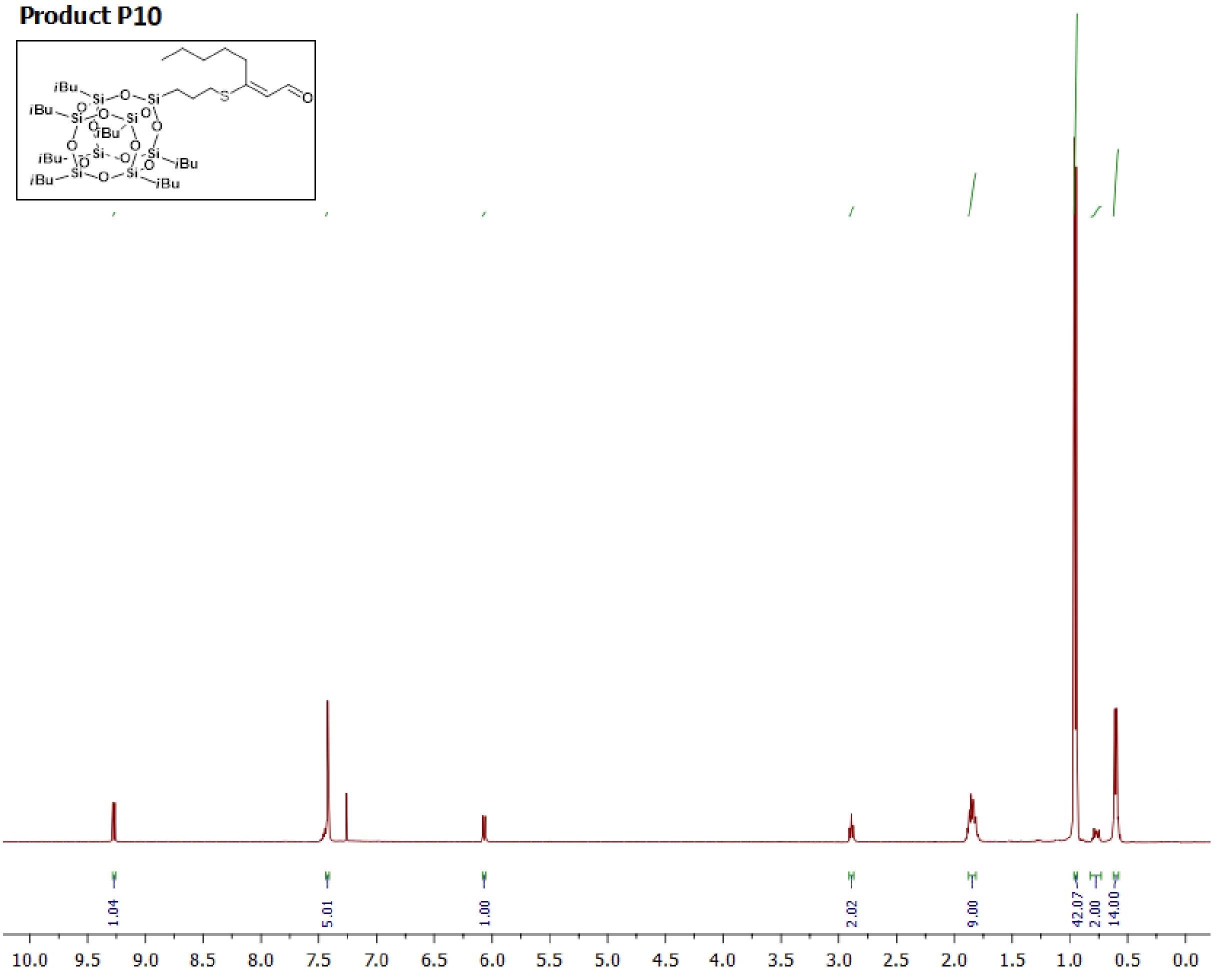

Figure S31. ^{1}H NMR (400 MHz, $CDCl_3$) of product **P10**

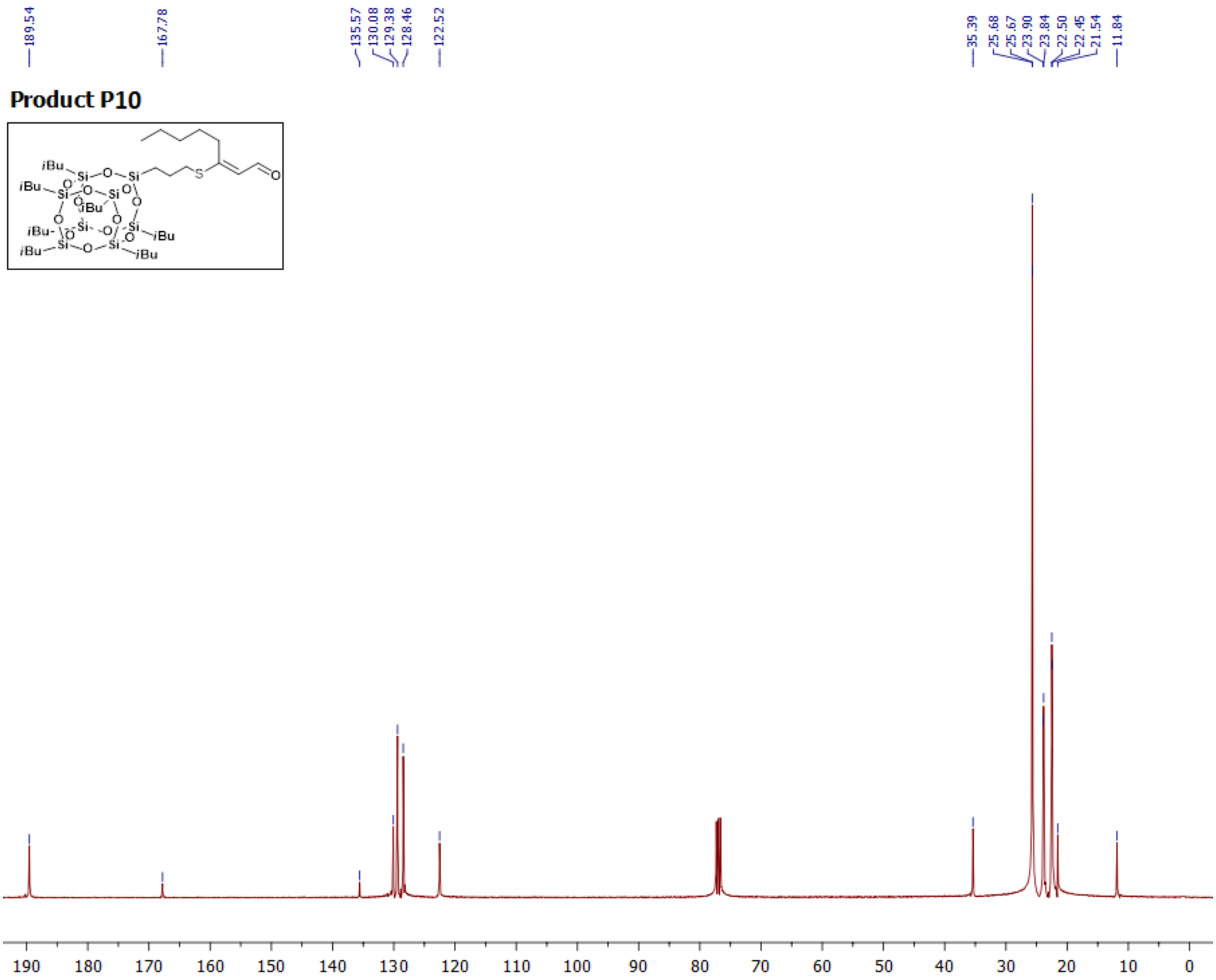

Figure S32. ^{13}C NMR (101 MHz, $CDCl_3$) of product **P10**

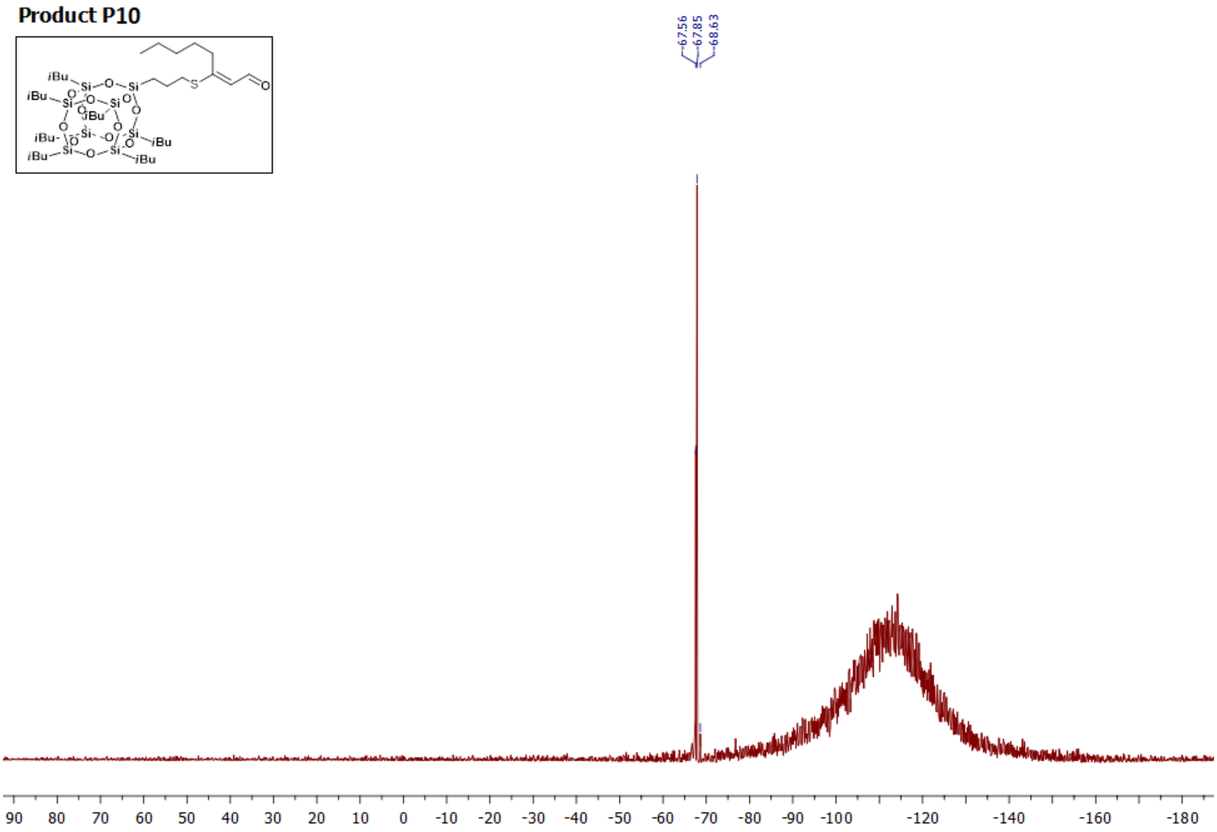

Figure S33. ^{29}Si NMR (79 MHz, $CDCl_3$) of product **P10**

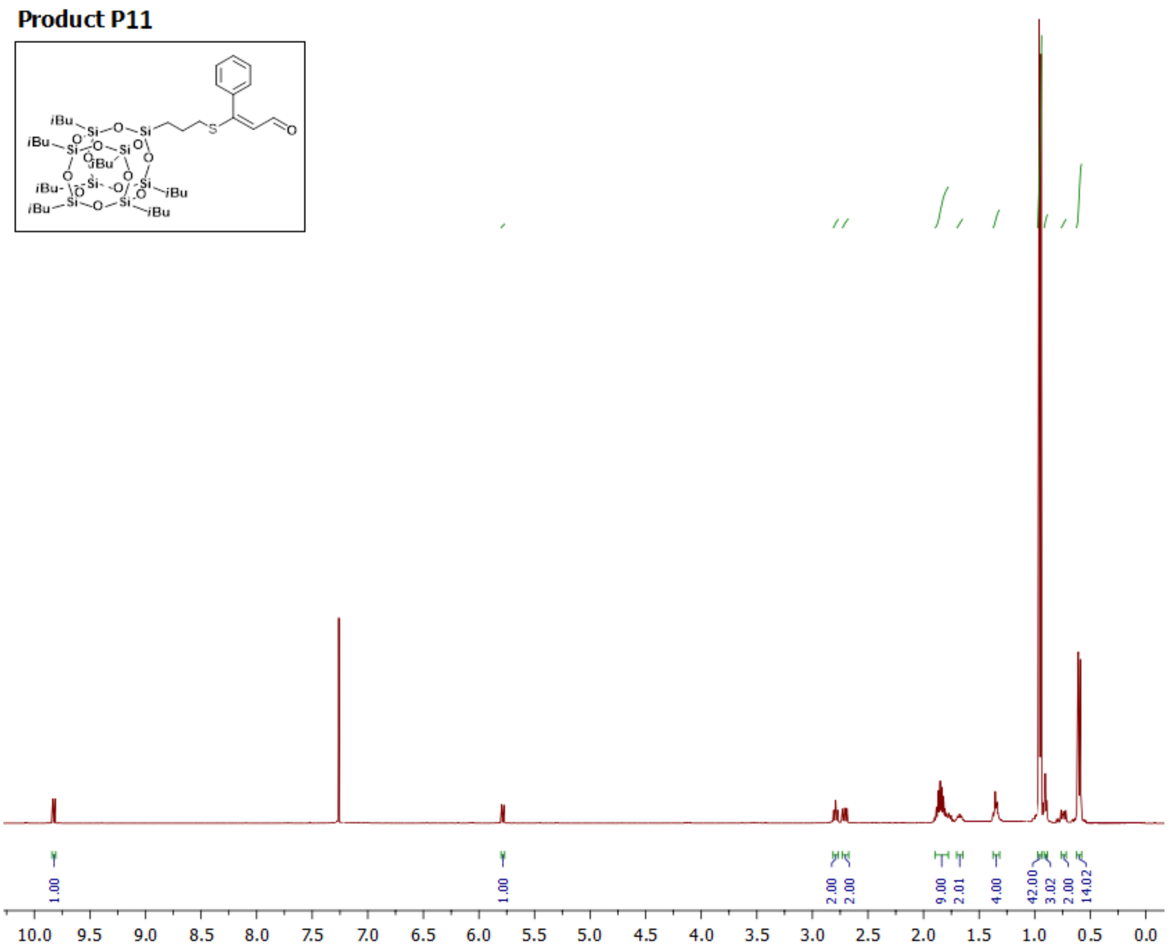

Figure S34. ^{1}H NMR (400 MHz, $CDCl_3$) of product **P11**

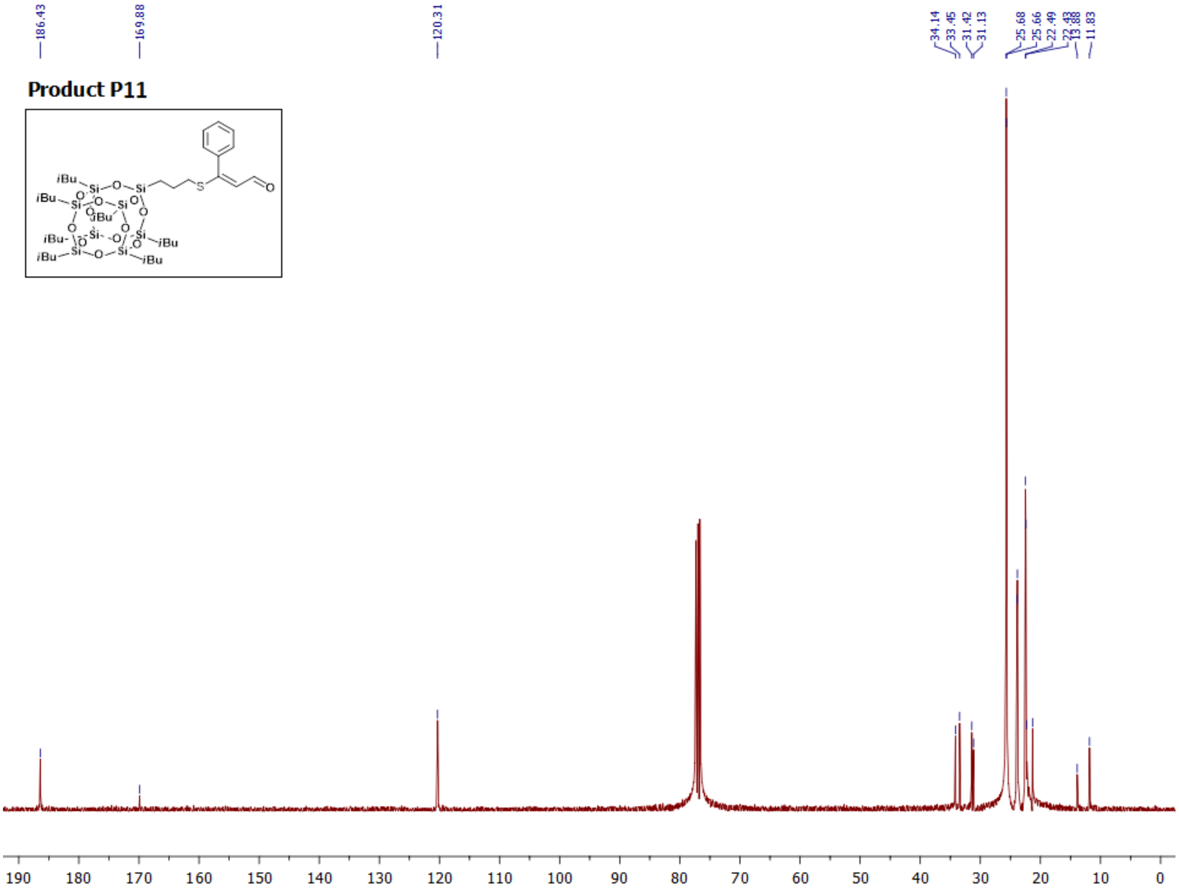

Figure S35. ^{13}C NMR (101 MHz, $CDCl_3$) of product **P11**

Product P11

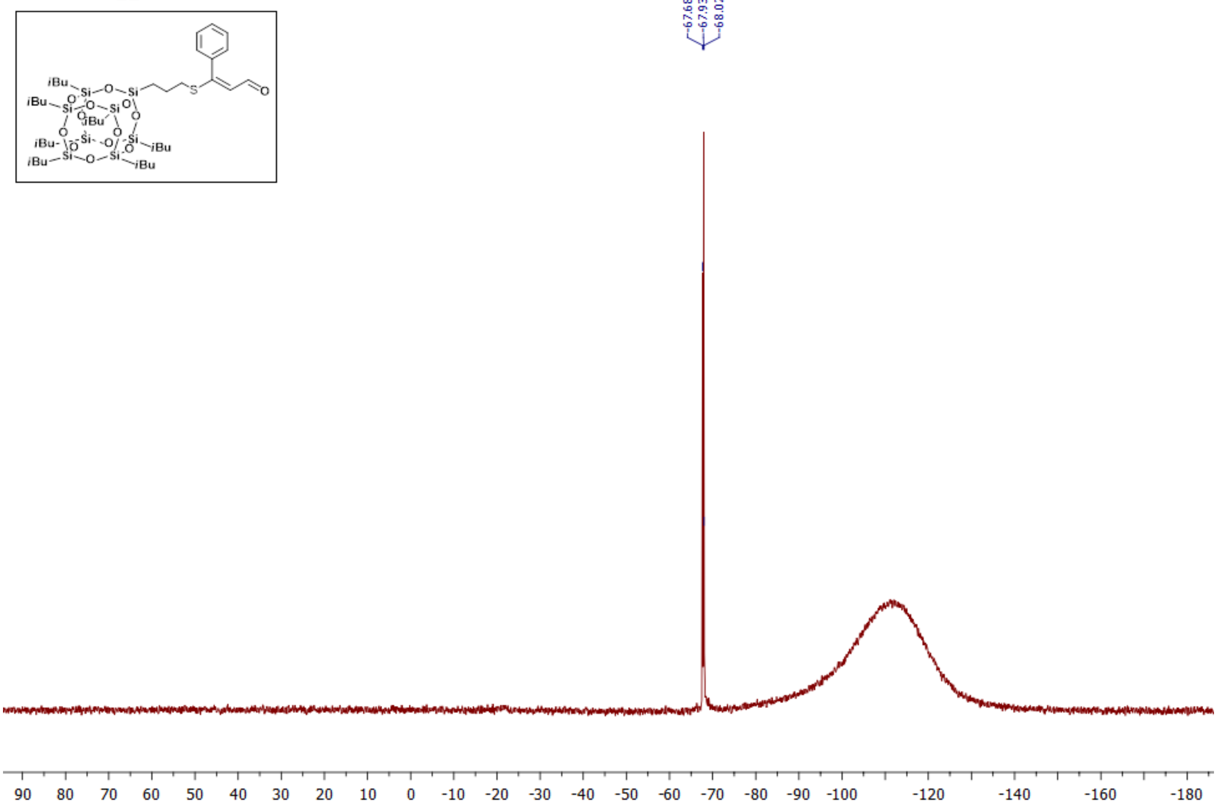

Figure S36. ^{29}Si NMR (79 MHz, $CDCl_3$) of product **P11**

Product P12

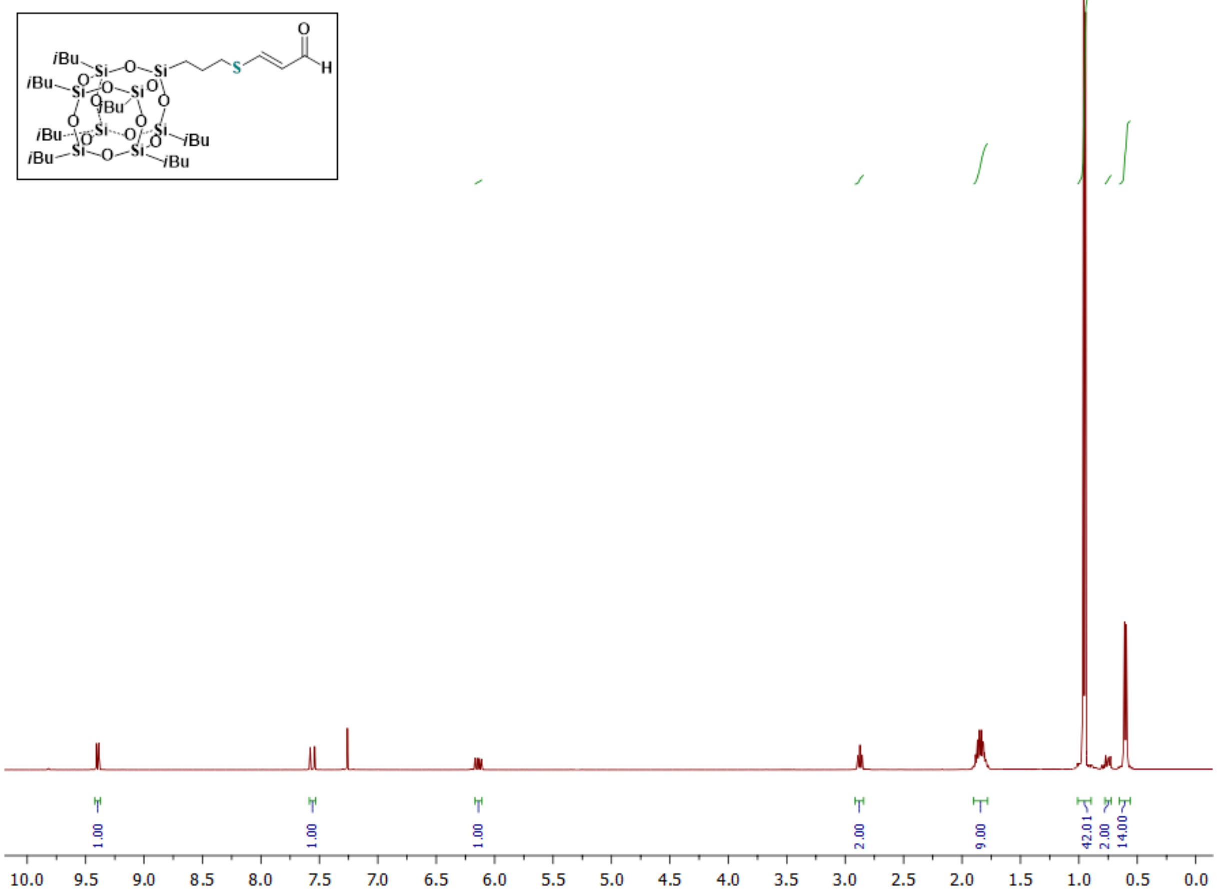

Figure S37. ^{1}H NMR (400 MHz, $CDCl_3$) of product **P12**

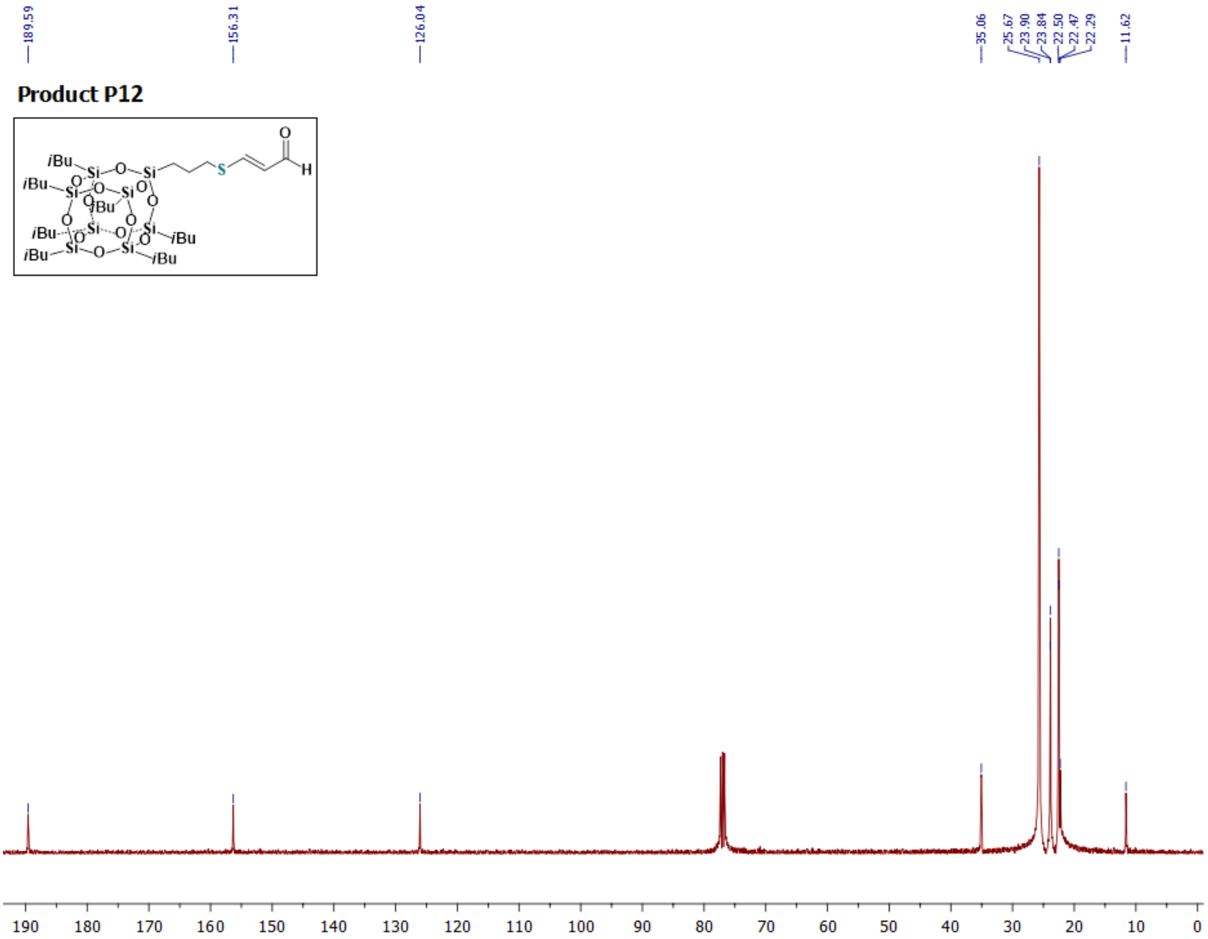

Figure S38. ^{13}C NMR (101 MHz, $CDCl_3$) of product **P12**

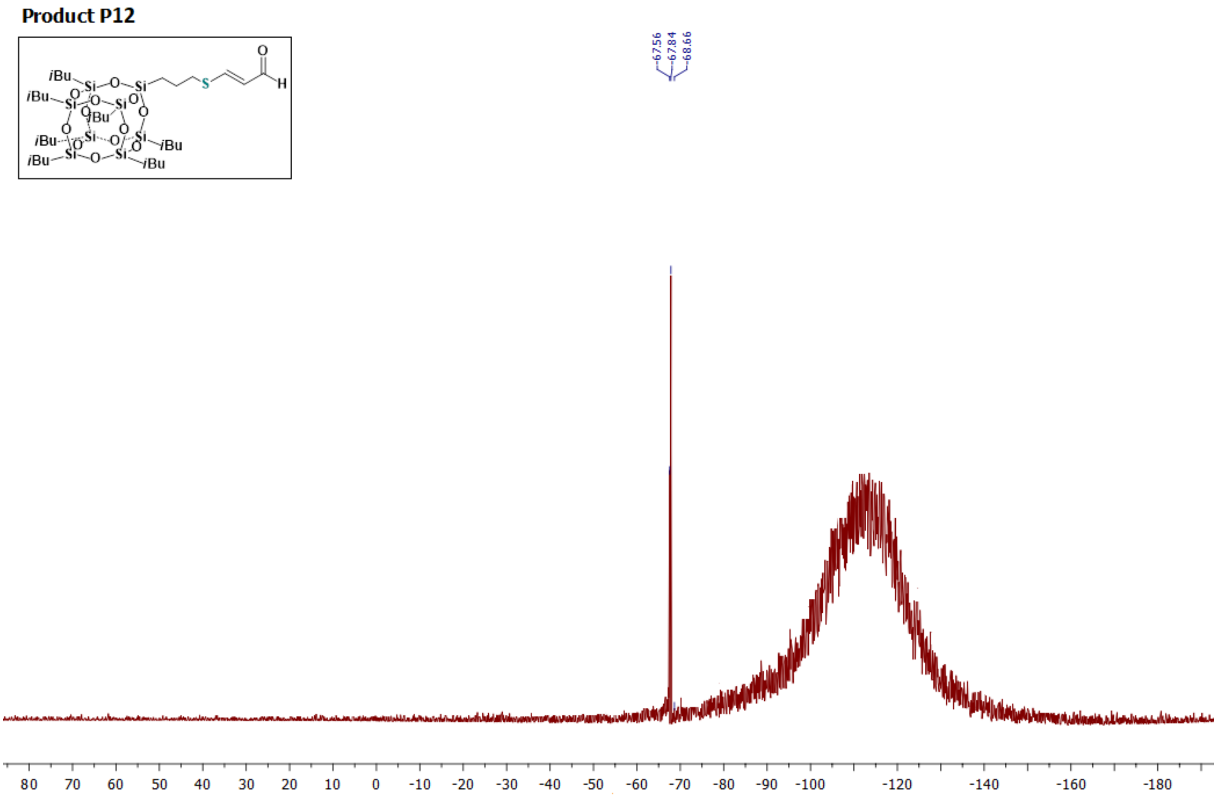

Figure S39. ^{29}Si NMR (79 MHz, $CDCl_3$) of product **P12**
